# Giant sulfur bacteria (Beggiatoaceae) from sediments underlying the Benguela upwelling system host diverse microbiomes

**DOI:** 10.1371/journal.pone.0258124

**Published:** 2021-11-24

**Authors:** Beverly E. Flood, Deon C. Louw, Anja K. Van der Plas, Jake V. Bailey

**Affiliations:** 1 Department of Earth and Environmental Sciences, University of Minnesota, Twin Cities, Minnesota, United States of America; 2 National Marine Information and Research Centre, Swakopmund, Namibia; The University of Akron, UNITED STATES

## Abstract

Due to their lithotrophic metabolisms, morphological complexity and conspicuous appearance, members of the Beggiatoaceae have been extensively studied for more than 100 years. These bacteria are known to be primarily sulfur-oxidizing autotrophs that commonly occur in dense mats at redox interfaces. Their large size and the presence of a mucous sheath allows these cells to serve as sites of attachment for communities of other microorganisms. But little is known about their individual niche preferences and attached microbiomes, particularly in marine environments, due to a paucity of cultivars and their prevalence in habitats that are difficult to access and study. Therefore, in this study, we compare Beggiatoaceae strain composition, community composition, and geochemical profiles collected from sulfidic sediments at four marine stations off the coast of Namibia. To elucidate community members that were directly attached and enriched in both filamentous Beggiatoaceae, namely *Ca*. Marithioploca spp. and *Ca*. Maribeggiatoa spp., as well as non-filamentous Beggiatoaceae, *Ca*. Thiomargarita spp., the Beggiatoaceae were pooled by morphotype for community analysis. The Beggiatoaceae samples collected from a highly sulfidic site were enriched in strains of sulfur-oxidizing Campylobacterota, that may promote a more hospitable setting for the Beggiatoaceae, which are known to have a lower tolerance for high sulfide to oxygen ratios. We found just a few host-specific associations with the motile filamentous morphotypes. Conversely, we detected 123 host specific enrichments with non-motile chain forming Beggiatoaceae. Potential metabolisms of the enriched strains include fermentation of host sheath material, syntrophic exchange of H_2_ and acetate, inorganic sulfur metabolism, and nitrite oxidation. Surprisingly, we did not detect any enrichments of anaerobic ammonium oxidizing bacteria as previously suggested and postulate that less well-studied anaerobic ammonium oxidation pathways may be occurring instead.

## Introduction

Sulfur cycling in marine sediments has been extensively studied and recently reviewed by Jorgensen et al., 2019 [1]. Sulfate, which is highly abundant in marine settings (~28 mM), is the most prevalent driver of the respiration of organic matter under anoxic conditions. The product of dissimilatory sulfate reduction, hydrogen sulfide is toxic to the aerobic respiratory pathway. But many bacteria have evolved to couple the oxidation of sulfide with aerobic respiration as well as other electron acceptors. Some of these sulfide-oxidizing bacteria are highly specialized and are dependent upon the oxidation of reduced sulfur (S_red_) species as their primary source of energy. The challenge for these specialists is that H_2_S is abiotically oxidized by O_2_ at fairly efficient rates. As such, they must develop mechanisms and/or relationships that enable them to have access to sources of both S_red_ electron donors and electron acceptors. One such group of sulfur-oxidizing specialists, who have developed some unique adaptations to exploit this lithotrophic niche, is the gammaproteobacterial family, Beggiatoaceae.

The family Beggiatoaceae is composed of 13 named genera of morphologically complex sulfur-oxidizing bacteria [2], some of which are the largest bacteria ever observed [3]. Until recently the Beggiatoaceae were classified as members of the Thiotrichales, which includes other morphologically complex sulfur-oxidizing bacteria such as *Thiothrix* and *Achromatium*. Phylogenic associations based on whole genome comparisons [4, 5] indicate that the Beggiatoaceae are distinctly different from other Thiotrichales, thus reviving the former order Beggiatoales, of which the Beggiatoaceae remain the sole family.

Members of the Beggiatoaceae are chemolithotrophs that often form dense mats in the surface layers of sulfidic sediments, reviewed by [6]. The Beggiatoaceae oxidize inorganic S_red_ species to intracellular stores of cyclooctasulfur (S_8_) [7, 8] (and polysulfides [9]) that can be further oxidized to SO_3_^2-^ and/or SO_4_^2-^ [10] or in the absence of extracellular terminal electron acceptors, can be respired to produce H_2_S [11–13]. In addition to growth fueled by S_red_ species, some members of the Beggiatoaceae utilize hydrogen as an electron donor under aerobic conditions and for maintenance energy when coupled to the reduction of stored S_8_ under anoxic conditions [14].

Most strains within the Beggiatoaceae are autotrophs that fix carbon via the Calvin-Benson-Bassham cycle [15] and perhaps in some cases, via the rTCA cycle as well [16, 17]. These autotrophs can also utilize some organic acids as carbon and/or as an energy source, but most have a limited capacity to grow on organic substrates [15, 18–20]. But there are exceptions in which inorganic carbon fixation is not a trait, particularly in freshwater strains which can be either chemolithoheterotrophs [21] or organoheterotrophs [22, 23]. Non-methane methylotrophy has also been reported in some members of the Beggiatoaceae [24, 25].

All of the Beggiatoaceae likely respire aerobically, but many if not most, prefer hypoxic or dysoxic conditions [20, 26–28]. In addition, certain members of the Beggiatoaceae can respire nitrate via the dissimilatory reduction to ammonia (DNRA) or via the denitrification pathway or both [29]. The largest members of the Beggiatoaceae achieve their large size due to a large central vacuole where high concentrations of NO_3_^-^ are stored [3, 30–33] and respired as needed [34]. The dynamics of inorganic nitrogen respiration within the vacuolate marine Beggiatoaceae is complex and not fully understood [16, 35]. Interestingly, nitrogen fixation has been shown to occur within the Beggiatoaceae under nitrogen-limiting conditions [36, 37] but nitrogen fixation has not been detected in vacuolated marine strains.

**Fig 1:**
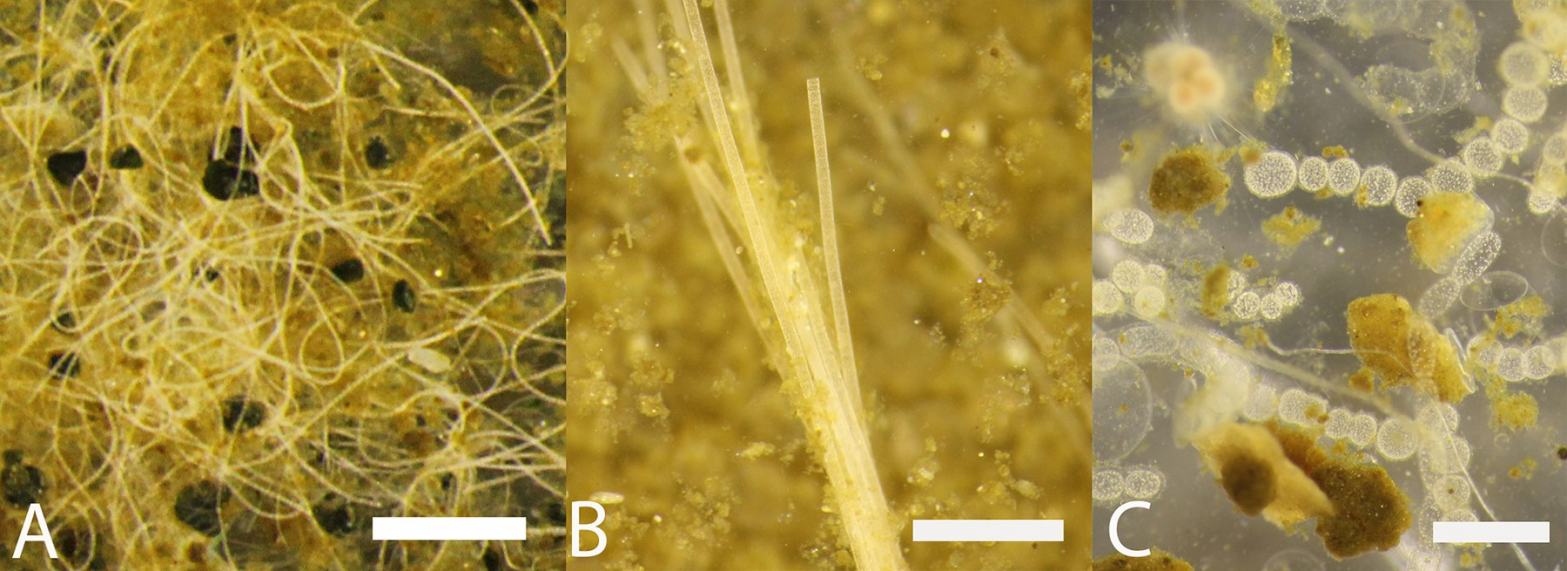
Light microscopy images of members of the Beggiatoaceae observed onboard the R/V mirabilis, offshore Namibia. A. Filamentous Beggiatoaceae often morphologically identified as “Beggiatoa”, B. Filamentous Beggiatoaceae often morphologically identified as “Thioploca”, C. Non-filamentous Beggiatoaceae often morphologically identified as “Thiomargarita”. Scale bars in A and B are 150 um, and C in 600 um.

In general, the Beggiatoaceae can be categorized into two morphological subgroups: filamentous and non-filamentous (coccoidal and chain-forming) strains, Fig 1. The morphology of the Beggiatoaceae is strongly linked to their ecology and responses to environmental conditions. Filamentous clades, i.e. *Beggiatoa* spp., *Candidatus* Maribeggiatoa spp., *Ca*. Isobeggiatoa spp., *Ca*. Parabeggiatoa spp., *Ca*. Allobeggiatoa spp., *Ca*. Halobeggiatoa spp., *Thioploca*, *Ca*. Marithioploca, *Ca*. Marithrix and *Thioflexithrix* spp., glide through the sediments to attractants such as H_2_S, and away from repellants, such as O_2_ [10, 26–28, 32] and light [38]_._ Coccoidal and chain-forming strains, *Ca*. Thiomargarita spp., *Ca*. Thiopilula spp. and *Ca*. Thiophysa spp., are non-motile and must resort to other mechanisms to access substrates and cope with stressful environmental conditions. Unfortunately, there are no coccoidal strains of the Beggiatoaceae in culture and few *in situ* studies [20, 28]. Strains of filamentous Beggiatoaceae cannot be rigorously distinguished from one another based on morphology alone [2], though gross morphological differences are characteristic of certain phylotypes. The same is true for that of the coccoidal chain-forming strains. The Beggiatoaceae can be distinguished from other bacteria based on their large size, general morphology, habitat and the presence of the intracellular sulfur globules.

Shelf sediments under the Benguela Upwelling System off the coast of Namibia, host representatives of most marine genera of the Beggiatoaceae. These sediments are rich in organic matter, primarily derived from phytodetritus resulting in a deposit of diatomaceous ooze. There is little terrigenous input except minor riverine input that is ephemeral and distal [39]. The upwelling of deep ocean waters promotes intensive primary productivity, fueling one of the richest fisheries in the world [40]. The deposition of the decaying pelagic community onto the shelf sediments results in robust microbial respiration and fermentation, which regenerates nutrients to the water column and further stimulates water column productivity [41, 42]. Under these high oxygen demand conditions, the benthic water column can be seasonally hypoxic or even anoxic [43, 44], harming benthic macro-communities [45, 46]. Sulfur-oxidizing bacteria, esp. members of the Beggiatoaceae, are thought to play a crucial role in detoxifying porewater sulfide fluxes into the water column [44] and are a likely food source for poorly characterized benthic biota [47]. Shallow sediments (< 200 m) can contain abundant Beggiatoaceae [3, 44, 48]. *Ca*. Thiomargarita spp. tend to occur in sediments less than ca. 150 m deep where high biological oxygen demand and fluxes of sulfide from the sediments result in bottom water anoxia. *Beggiatoa* and other filamentous strains tend to be found in sediments where bottom waters are hypoxic but not anoxic. Other factors governing ecological zonation of the Beggiatoaceae remain unresolved. Studies of niche partitioning and habitat selection by members of Beggiatoaceae suggest that carbon sources and the ability to store intermediates such as sulfur may play a role [49], as well as the stability of redox gradients [3, 50], differences in growth rates and the absence or presence of nitrate vacuoles [44].

Little is known about the interactions between representatives of the Beggiatoaceae and co-occurring macro- and microbiota. Numerous observations of Beggiatoaceae attaching to marine animals have been made at methane seeps [51, 52] and in marine sediments [53]. Likewise, motile and filamentous Beggiatoaceae are thought to interact with other microorganisms. For example, Beggiatoaceae-dominated mats in marine sediments increase advective flow and produce ammonium and sulfate that are thought to stimulate sulfate reduction and anaerobic ammonium oxidation [54]. Nutrient exchange between sulfate-reducing bacteria and filamentous Beggiatoaceae has also been previously proposed [55, 56]. Some interactions between representatives of the Beggiatoaceae and smaller bacteria may involve physical contact because the large size and production of mucous sheath material can provide a habitat for epibiont bacteria. For example, the sheath of filamentous Beggiatoaceae have been shown to be populated by ammonia oxidizing archaea [57] and annamox bacteria [58]. No molecular studies have been performed on non-filamentous Beggiatoaceae, but bacteria have been previously noted attached to the exteriors of Thiomargarita spp. [59].

In this study, we employed iTag sequencing of the 16S rRNA gene comparing communities associated with pooled filamentous Beggiatoaceae, as well as pooled non-filamentous Beggiatoaceae, and compared these communities with those of their host sediments collected from the continental shelf off of Namibia in southwestern Africa. We hypothesized that intermediates/end products from both sulfur oxidation and nitrate reduction would promote sulfate-reducing and ammonium-oxidizing strains to be significantly associated with both filamentous and non-filamentous Beggiatoaceae, similar to that as previously described in other environments. In addition, we predicted that the comparison of geochemical profiles collected from these sediments with that of the composition of the microbial communities would provide insights into niche partitioning by members of the Beggiatoaceae in these sediments.

## Methods

### Sample acquisition, in situ geochemistry, and sample preservation

Namibian Ministries of Fisheries and Marine Resources approved and supervised sample collection for this study and two scientists who are representatives of the NMFMR are co-authors on the manuscript. An independent field permit was not required. Sediment cores were collected on an April 20–27 2017 expedition of the R/V *Mirabilis* via a MC- 200–4 or MC-400 multi-corer. Samples were collected from four marine stations: Station 23020 (-23.00125, 14.04775; depth 125 m), Station 23002 (-22.999967, 14.3708; depth 36.4 m) Station 20020 (-20.002283, 12.67975; depth 85.8 m), and Station Geochem 2_1 (-24.050,733, 14.250233; depth 118 m). Bulk water column dissolved O_2_ concentrations were measured via Winkler Titration [60]. Dissolved O_2_ concentrations in waters above the sediment was measured with a ISO2 Dissolved Oxygen Meter (World Precision Instruments, Sarasota, USA).

Porewater samples were acquired at 1 cm scale horizons from sediment cores collected from Stations 23020, 23002, 20020 but not Geochem2_1. Rhizon CSS samplers (Rhizosphere, Netherlands) filtered the porewater into anoxic Vacutainer® tubes (BD, Franklin Lakes, NJ). Porewater samples, collected to quantify sulfide, were acidified and stored with ZnCl_2_ (0.5% w/v) to preserve the samples. Porewater samples were stored at 4°C until analyzed. The sediment cores were immediately sectioned. Subsectioned samples were preserved in DNA/RNA Shield™ (Zymo Research Corp., Irvine, CA) and were stored at -20°C during transport. A portion of the top 3 cm of each core and overhead seawater, referred to as “host sediments” was removed and stored at 4°C for collection of filamentous and non-filamentous Beggiatoaceae. Then the host sediments were examined shipboard for the presence of Beggiatoaceae using a Zeiss Primovert inverted microscope. The Beggiatoaceae filaments and chains were rinsed in 0.2 μm filtered seawater and then they were pooled using a micropipette based on morphology, i.e., filamentous samples and non-filamentous samples, until an assemblage of ~100 filaments or coccoidal chains was achieved. Then the Beggiatoaceae samples and the host sediment from which they were removed were stored separately in DNA/RNA Shield™ at -20°C. Additionally, upon completion of the collection of Beggiatoaceae samples, the rinse water was preserved in DNA/RNA Shield™ and processed alongside the biological samples as a negative control.

### Porewater analyses

Porewater samples were diluted 100-fold and ran in triplicate for ion chromatography analysis. Major porewater cations (Li^+^, Na^+^, NH_4_^+^, K^+^, Mg^2+^, and Ca^2+^) were quantified on a Dionex™ ICS 5000+ Capillary HPIC™ system equipped with a CS16-4μm (3 x 250 mm) column and a CERS™ 500 (2 mm) suppressor with an external water regenerant flow and 20 mM methanesulfonic acid as the eluent. Porewater anions (F^-^, acetate, formate, Cl^-^, NO_2_^-^, Br^-^, NO_3_^-^, SO_4_^2-^, S_2_O_3_^2-^, and PO_4_^-^) were analyzed on the same instrument equipped with a Dionex™ IonPac™ AS19-4μm (2 x 250 mm) column, an AERS™ 500 (2 mm) suppressor, external water regenerant flow and an EG III NaOH eluent generator cartridge. H_2_S was measured in triplicate via the methylene blue assay [61] (Product # 2244500; Hach, Loveland, CO).

### iTag analyses

DNA extractions were performed in a LabConco A2 biological safety cabinet using the ZymoBIOMICS DNA miniprep kit (Zymo, Irvine, CA). The University of Minnesota Genomics Center generated iTag libraries of the V4 hypervariable region as previously described [62]. The V4 primers used were 515F GTGYCAGCMGCCGCGGTAA [63] and 806R GGACTACHVGGGTWTCTAAT [64]. The Beggiatoaceae samples’ rinse waters, and two UMGC water controls were sequenced on one lane of MiSeq for paired-end 2X300 bp reads. Cutadapt v. 2.10 was employed to remove primers and adapters [65]. The paired end reads were assembled using DADA2 v1.16.0 [66] with a maximum expected error rate of 2. phiX was removed prior to error detection, merging of pairs and chimera detection. Taxonomic assignment was performed using the Silva database v. 138 [67]. Bioinformatic and statistical analyses was performed using tools in PhyloSeq 1.32.0 [68]. R package Decontam 1.8.0 [69] identified contaminating amplicon sequence variants (ASVs). Seven ASVs of the genera *Chryseobacterium*, *Lysinibacillus*, *Bradyrhizobium*, *Cutibacterium*, *Stenotrophomonas*, and unclassified members of the clades Xanthomonadaceae and Desulfobulbales were classified as contaminants and were bioinformatically removed from analyses. Post decontamination, the control samples and four samples of low abundance (<15,000 ASVs) were removed prior to further analysis within PhyloSeq. Normalization via variance stabilization transformation (subsampling), as well as detection of ASVs with statistically different abundances, were performed with DESeq2 v. 1.28.0 [70]. The DESeq2 test and parameters for detecting statistically different abundances employed a Wald test with a parametric fit, Cook’s distance filtering, and alpha (minimal P-value) set to 0.01. ASVs DESeq2 selected as unclassified at the phylum level were queried (blastN) against the National Center for Biotechnology Information (NCBI) non-redundant nucleotide collection. The top scoring accession was downloaded and queried alongside the unclassified ASVs against the Silva database using the SINA aligner 1.2.11 and were aligned and classified with a rejection below 70% identity. The SINA aligner generated a neighbor joining tree via RAxML using the GTR model and Gamma rate model for likelihoods. The tree was beautified in Figtree v.1.4.4. Additionally, Adobe Illustrator 2020 was used to beautify all images and to generate the modified Venn Diagram.

## Results and discussion

**Fig 2:**
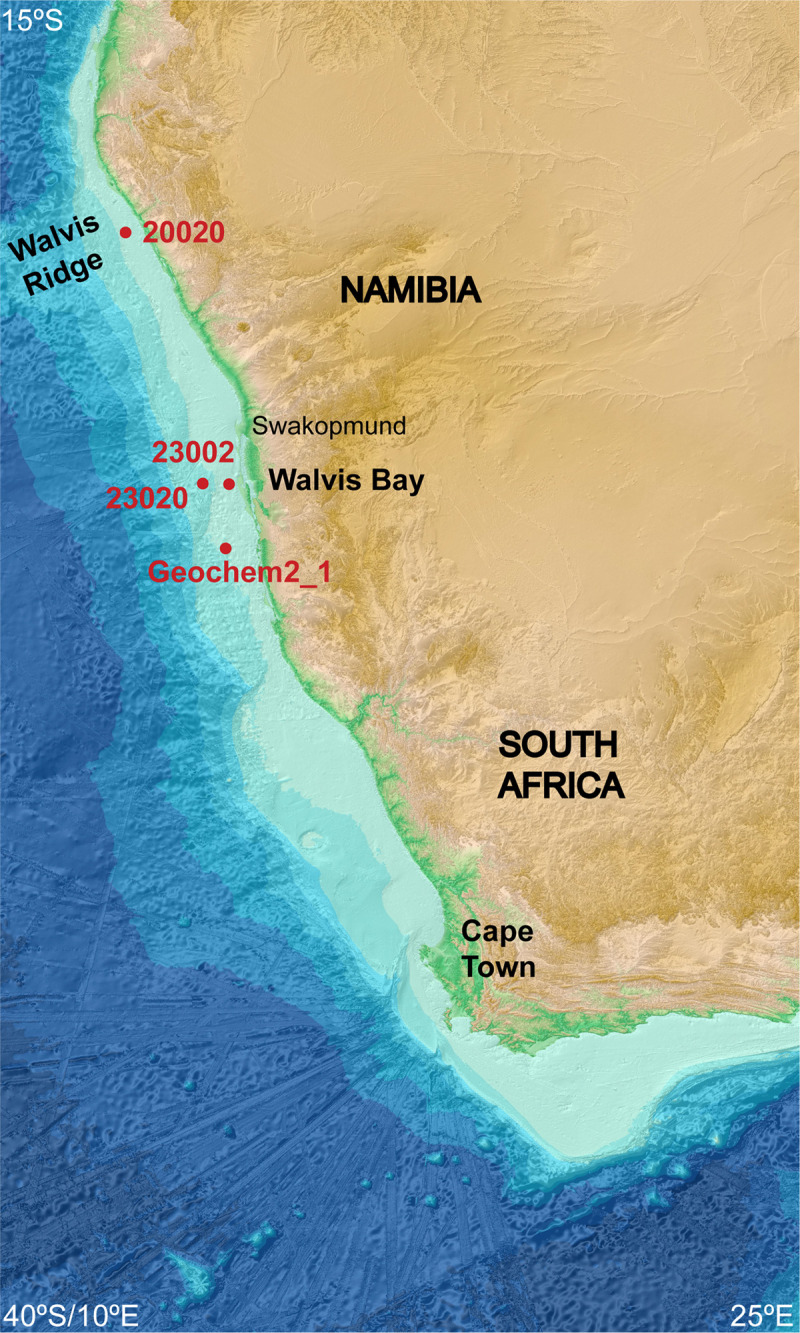
Map of sampling stations off the coast of Namibia, Africa. The sampling sites in this study were Marine Station 23002 (-22.999967, 14.3708), depth 36.4 m; Station 23020 (-23.00125, 14.04775), depth 125 m; Station 20020 (-20.002283, 12.67975), depth 85.8 m; and Station Geochem2_1 (-24.050,733, 14.250233), depth 118 m. The baseline map was reproduced from the GEBCO world map 2021, www.gebco.net.

**Fig 3:**
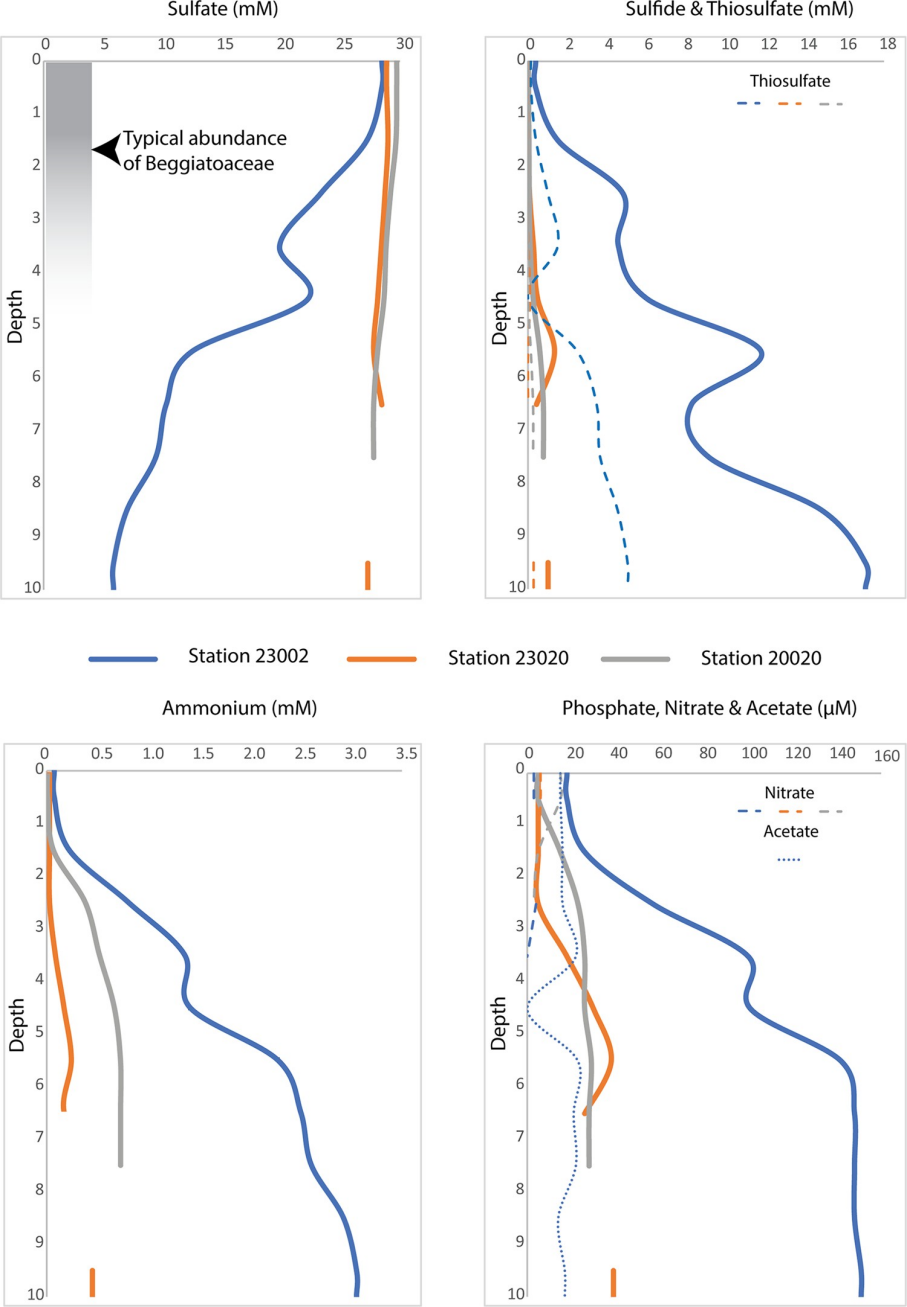
Geochemical profiles for the top 10 cm of sediment core porewaters with general zonation of Beggiatoaceae. Porewaters were collected from marine stations 23002 (blue; 1–10 cm), 23020 (orange; 1–7, 9–10 cm) and 20020 (grey; 1–7 cm). Formate (<11 μM) and nitrite (<2 μM) and other data points not shown for analyzed 1cm horizons were below detection limits. Standard deviations were low and representative crossbars were not visible on plots.

**Fig 4:**
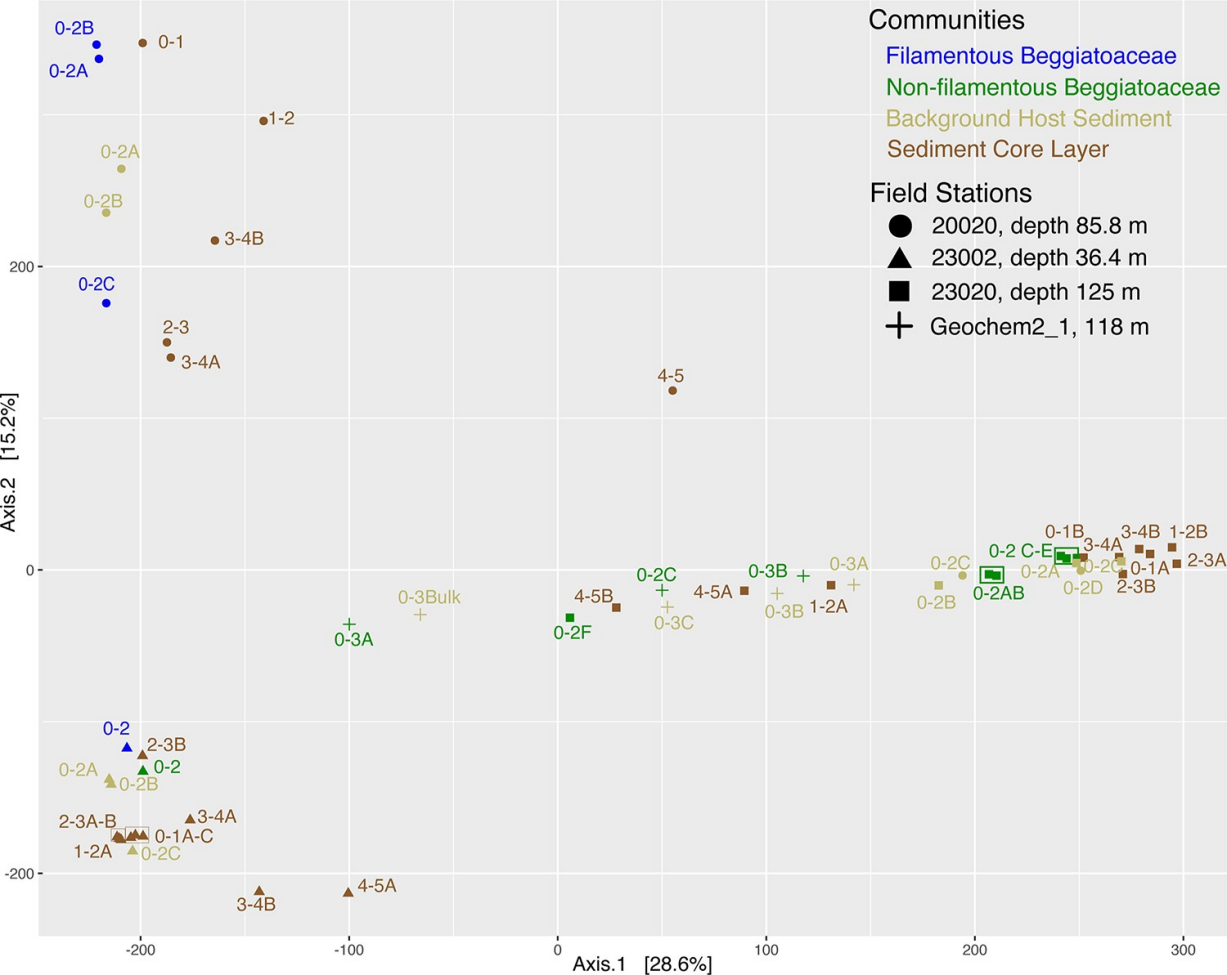
Principal coordinate analysis of 16S rRNA communities of samples collected from four marine stations: 23002, 20020, 23020 and Geochem2_1 and at depths between 0–5 cm. The plot was generated using multidimensional scaling with Euclidean distancing.

We collected sediment samples from four marine stations- Station 23002 (36.4 m deep), Station 20020 (85 m), Station GeoChem2_1 (118 m) and Station 23020 (125 m), Fig 2. Both O_2_ profiles of the water columns (S1 Fig) and microprofiling of the bottom water above the cores indicate that all stations were either hypoxic or anoxic. Stations 23002 and 23020 are the closest stations to one another along the -23 latitude axis but geochemical profiles, Fig 3, and Principal Coordinate Analysis, Fig 4, as well as hierarchical clustering via Euclidean distance matrix (S2 Fig) indicate that the two sampling stations at deeper water depths were more similar to one another, while the other sites were distinctly different. Measures of richness, Chao1 and Shannon diversity index, indicate that communities from the deepest station, Station 23020, had the greatest diversity and richness, S3 Fig, while communities from the shallowest station, Station 23002 were the least diverse. Despite normalization, differences in DNA library sizes likely affected many of these analyses whereby some samples were sequenced at a depth that provided substantial characterization of the microbial community, but a few samples were not, S4 Fig.

### Both morphotypes occur at shallow Station 23002

Station 23002 is found within the diatomaceous mud belt [71] but it lies much closer inshore than that of the other mud belt stations and thus likely encounters greater terrigenous input. This is only station where we were able to collect samples of both filamentous and non-filamentous Beggiatoaceae bacteria. But bacteria from both groups of Beggiatoaceae morphotypes were not abundant at this inshore station, thus we were only able to collect one pooled sample of each morphotype from the top 3 cm of the sediment core. The Beggiatoaceae ASVs in the filamentous samples were predominantly the candidate strains *Ca*. Marithioploca, *Ca*. Maribeggiatoa and *Ca*. Parabeggiatoa. These ASVs were also detected in the sediment core with abundances trailing off at or above a depth of 3 cm beneath the sediment/water interface. Other Beggiatoaceae ASVs were also detected in the filamentous sample including *Thioflexithrix* and unclassified Beggiatoaceae ASVs. To the best of our knowledge, this is the first report of a *Thioflexithrix* from a marine environment. The recently discovered type strain, *T*. *psekupsensis* D3, was isolated from a thermal sulfidic spring [37]. The non-filamentous sample contained a mix of *Ca*. Thiomargarita nelsonii, *T*. *namibiensis*, *Ca*. Marithioploca, *Ca*. Maribeggiatoa, and an unclassified ASV. ASVs of *Ca*. Thiomargarita were barely detected in the sediment core. But *Thiomargarita’s* sister taxa, *Ca*. Thiopilula, was detected throughout the top 5 cm of sediment core. We were not surprised by the relatively low abundance of Beggiatoaceae ASVs in the core samples and even in the pooled samples. Indeed, low DNA amplification of Beggiatoaceae has been previously observed in these sediments [72]. Many marine strains have introns within their 16S rRNA gene [73] which are believed to be inhibitory to PCR amplification, (Salman-Carvalho *personal comm*. and our own experiences).

**Fig 5:**
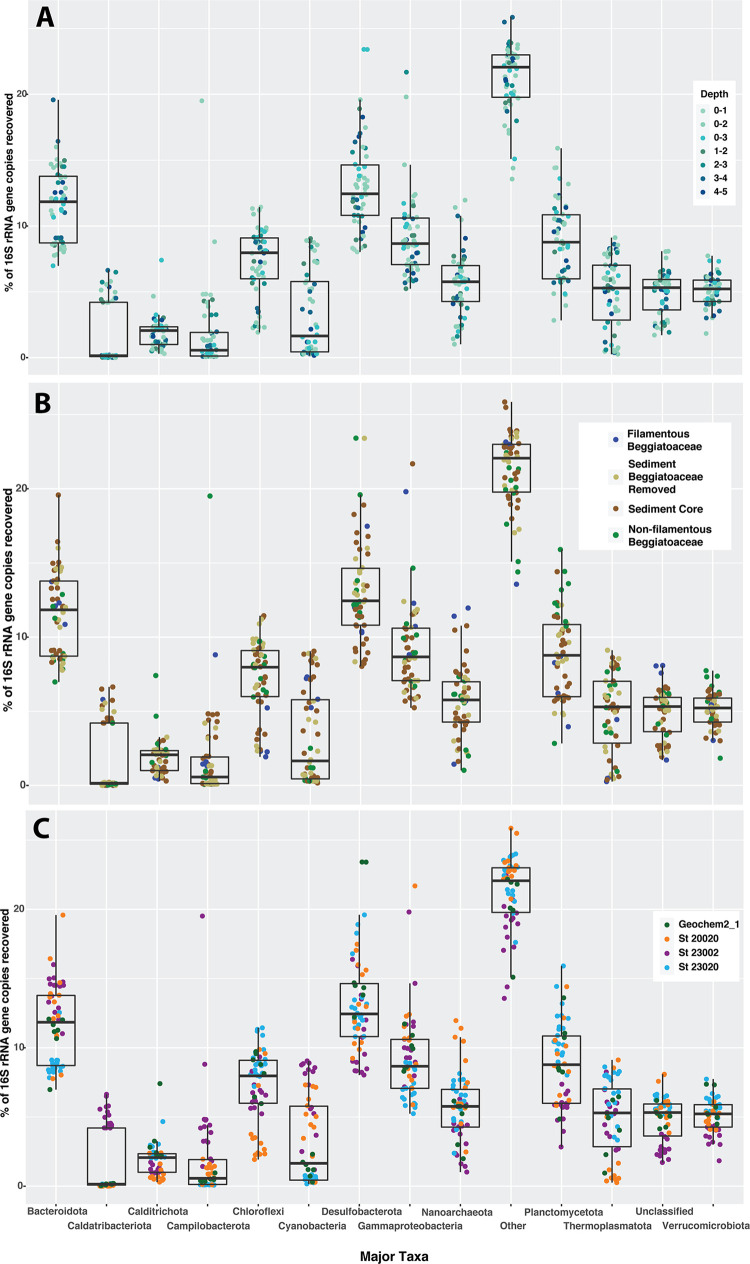
Box plots of the phyla of ASVs by percent community composition by A. depth in cm. B. sample type. C. location.

Geochemical profiles of the top 10 cm of the sediment core collected from Station 23002 suggest that these sediments are experiencing extensive sulfate reduction near the sediment/water interface Fig 3. Shifts in dominant taxa that may be associated with major metabolic regimes were evident at discreet horizons. Box plots of the most abundant phyla and proteobacterial classes, Fig 5, indicate that the community composition of the top 5 cm of the sediment cores contained a larger fraction of Caldatribacteriota, Cyanobacteria (predominantly diatom chloroplasts but free-living Cyanobacteria cannot be ruled out [74]) and Campilobacterota (formerly known as Epsilonproteobacteria) than other cores. The Verrucomicrobiota and ASVs unclassified at the phylum level composed a smaller fraction of the core community than the other marine stations. Caldatribacteriota is a newly named phylum that includes the former candidate phylum Atribacteria (OP9/JS1), which are thought to be primarily fermenters [75] that may have a syntrophic metabolism with methanogens [76] and that are commonly found in abundance in organic-rich anoxic marine sediments. Fermentation, such as that carried out by representatives of the Caldatribacteriota, is potentially the primary metabolism responsible for the increase in ammonium at depth [77] along with dissimilatory reduction of nitrate [78, 79]. Between 4–5 cm sediment depth, 1 mM of ammonium was consumed concomitantly with thiosulfate and sulfide, generating an increase in sulfate. Around 7 cm of depth, there was a significant drawdown of sulfate. A previous study of nearby sediments indicate that drawdown of sulfate is coupled to AOM [44]. We did not perform iTag sequencing on sediments below 5 cm. But ASVs of ANME-1a, ANME-1b and ANME-2c archaeal anaerobic methylotrophs were present throughout the top 5 cm of the core along with abundant and diverse archaeal methanogens.

One reasonable interpretation of these geochemical profiles is that the horizon at 4–5 cm reflects intense anammox activity coupled to sulfur oxidation and nitrate reduction [58, 80]. However, our iTag data indicated that the anammox bacteria in these sediments, *Ca*. Scalindua spp., were present primarily in the top 3 cm and were almost absent between 4–5 cm of depth. Since anammox bacterial abundance has been shown to be directly correlated to anammox activity [81], it seems likely that other processes were removing NH_4_^+^ at this horizon. However, the iTag community profiles provided little insight into the origin of the drawn down of NH_4_^+^ at this horizon. For an in-depth analysis and discussion, please see S1 Text.

### Only filamentous Beggiatoaceae occur at Station 20020

Marine Station 20020 lies outside of the diatomaceous mud belt. The Beggiatoaceae in the filamentous samples were less diverse than those found at Station 23002 and non-filamentous Beggiatoaceae were barely detectable in these sediments. The dominant Beggiatoaceae ASV was also the most abundant at Station 23002, an ASV of *Ca*. Marithioploca. The second most abundant ASV at Station 20020 was an unclassified member of the Beggiatoaceae. Porewater chemistry from the top 7 cm of the core collected from Station 20020 indicated that the top cm of the core was likely hypoxic while low rates of sulfate reduction, fermentation and DNRA occurred below. There was a notable shift in community composition between the 4–5 cm horizon, where *Ca*. Marithioploca was not detected. Two phyla in the top 4 cm, Chloroflexi and Thermoplasmatota, represented a smaller percent of community abundance in comparison to other stations, Fig 4. The Chloroflexi found at the other field stations were predominantly within the Anaerolineales, which are facultative or strict anaerobes that ferment a broad range of substrates [82]. Thermoplasmatota is a new phylum composed of many previously uncharacterized clades [83]. The most notable difference between the sediments here vs. other stations was the absence of one the most abundant ASVs (ASV #4) in our entire dataset, which was classified as a member of Marine Benthic Group D and DHVEG-1. Representatives of this clade are known to be restricted to anoxic environments [84]. These sediments had an elevated abundance of “cyanobacteria” but again top blastN scores indicate that these ASVs may be plastids of benthic foraminifera, esp. *Virgulinella fragilis*. Perhaps bioturbation generated by vertical migration of filamentous Beggiatoaceae and benthic foraminifera coupled with reduced organic detrital input resulted in the upper 4 cm of sediment being dysoxic, and not anoxic. *Ca*. Marithioploca spp. from the Peruvian margin are thought to be transient in nature occurring in sediments transitioning from hypoxic to anoxic conditions brought about by seasonal and other climatic changes [85, 86].

### Non-filamentous Beggiatoaceae occur in mud belt stations

Initial studies concerning *Ca*. Thiomargarita niche preferences concluded that *Ca*. Thiomargarita abundances were significant when the sediments were euxinic and there was a high flux of sulfide into the water column [48]. A subsequent study monitored four marine stations over a period of three years, measuing both sulfide and methane concentrations as well as the number of *Ca*. Thiomargarita and concluded that there was no correlation between *Ca*. Thiomargarita abundances with porewater and bottom water chemistry [87]. In our study, non-filamentous Beggiatoaceae were very abundant at Marine Station 23020. We were able to collect six replicate samples of ~100 chains each. The two dominant Beggiatoaceae ASVs were *Thiomargarita namibiensis* and *Ca*. T. nelsonii. In much lower abundances were strains of *Ca*. Thiopilula and unclassified Beggiatoaceae.

Marine Station 23020 is in the heart of the diatomaceous mud belt. Total organic carbon can exceed 10 wt.% in these sediments [39]. Our geochemical porewater profiles of sulfate, Fig 2, indicate that Marine Station 23020 was more anoxic in comparison to Marine Station 20020. Yet these organic-rich sediments had low levels of sulfide accumulation and sulfate drawdown, potentially indicating that limited sulfate reduction and sulfide oxidation was occurring in these sediments. However, an alternative interpretation is that the rates of both metabolisms were roughly equal, a scenario has been observed at nearby shelf sediments containing *Ca*. Thiomargarita (23°46.520 S, 014°17.960E, 110 m water depth) [88].

Sediment pore water horizons that are characterized by high levels of inorganic phosphate, which are typically found below the sediments containing *Ca*. Thiomargarita spp. [89], are less enriched in phosphate in our samples. It is certainly possible that less inorganic phosphate reaches these sediments than in some other sediments with abundant *Ca*. Thiomargarita spp. Or that the phosphate was sequestered as polyphosphate within the cells at the time of collection. Another possible explanation is that these sediments have undergone methane gas ebullition in the recent past resulting in suspension of sediments and the mixing of porewaters with bottom waters. Periodic suspension of host sediments has been proposed to be the primary mechanism for *Ca*. Thiomargarita to replenish its nitrate stores [3]. Indeed, large methane gas pockets were detected in 2004 in the region of Marine Station 23020 [90]. Alternatively, perhaps these sediments were disrupted by bioturbation but potential bioturbators were not observed and these frequently anoxic sediments are typically depauperate in benthic invertebrates [91].

At Marine Station GeoChem2_1, a sediment core was not collected for geochemical profiling and characterizing community composition 1 cm horizons. Instead, the top 0–3 cm of surface sediments collected for three pooled non-filamentous Beggiatoaceae samples for comparison with their host sediments and one unaltered “bulk” sample was retained for comparison. These shelly phosphatic sand sediments are well characterized by previous studies (Marine Stations 229 and 12810) [92, 93]. Porewater phosphate concentrations can reach 0.5 mM [94]. *T*. *namibiensis* and *Ca*. T. nelsonii were roughly equal in concentration in the non-filamentous Beggiatoaceae samples of station GeoChem2_1. We also recovered some *Ca*. Maribeggiatoa and *Ca*. Thiopilula in the non-filamentous Beggiatoaceae samples.

Euclidean clustering of samples from all communities resulted in community ASV compositions of Marine Station 23020 and GeoChem2_1 being more similar to one another than to the other two stations, S2 Fig. In general, at Marine Station 23020 the Bacteroidota, Campilobacterota, Cyanobacteria, and Gammaproteobacteria comprised less of the community than other marine stations. Sediments from this location were in general more diverse than stations where filamentous Beggiatoaceae were present, S3 Fig.

### ASVs uniquely associated with filamentous Beggiatoaceae

We collected three samples of ~100 chains of filamentous bacteria from Marine Station 20020 but only one sample from the shallow Station 23002. Thus, we were able to employ DESeq2, S1 Table, to detect statistically significant associations of ASVs with the filamentous Beggiatoaceae at Station 20020 but not Station 23002. DESeq2 is an R package that was original designed for detecting differential expression between two treatments in RNA sequencing experiments and accounts for differences in sample library sizes. Significant associations were determined through a Wald’s Test (p-value > 0.01) adjusted for potential false discoveries (padj). DESeq2 did not identify an ASV in any significant abundance (>40 counts) that was positively correlated with the filamentous Beggiatoaceae samples vs. their host sediments at Station 20020. One of three filamentous Beggiatoaceae samples from Station 20020 had a significantly smaller library size (30,000 vs > 250,000), S4 Fig, which we believe affected the results of the analysis and that there were other likely significant associations to be found. Examples include two strains of the Crenarchaeota *Ca*. Nitrosopumilus which were enriched in the two Station 20020 samples with greater sequencing depth (> 800; >100) vs. their host sediments (< 225; <60). This host-microbe association has been observed with *Ca*. Maribeggiatoa of the hydrothermal system of the Guaymas Basin, Gulf of California [57]. Archaeal aerobic ammonia-oxidizers, such as *Ca*. Nitrosopumilus, can tolerate very low oxygen conditions [95], have high rates of activity [96], and were present in all stations in this study, but were particularly abundant in the top 0–2 cm. But unlike samples from Station 20020, no enrichment was evident in the filamentous Beggiatoaceae sample from Station 23002. Instead, a different aerobic ammonia-oxidizer in low abundance was enriched in this Beggiatoaceae sample, a Nitrosococcaceae SZB85 (49 vs. 0). Members of the Nitrosococcaceae can couple denitrification with sulfide-oxidation under anoxic conditions [97]. Their association with motile Beggiatoaceae would be highly beneficial to both the epibiont and host under hypoxic conditions, but potentially competitive under anaerobic conditions.

A host specific relationship between the filamentous sulfate-reducer *Desulfonema*, and *Ca*. Marithioploca was previously identified in Chilean sediment samples [98] as well as with “Beggiatoa” of the Frasassi Cave System in Italy [56]. Also, significant horizontal gene transfer between the Beggiatoaceae and a strain of *Desulfonema* has been suggested [99]. We found that the filamentous Beggiatoaceae sample from shallow Station 23002 had an ASV classified as *Desulfonema* that was more abundant than that ASV in the host sediments (117 vs. ~5). ASVs of *Desulfonema* were abundant in Beggiatoaceae samples from Station 20020, but not more so than the host sediments. Nor did we detect a potential specific association between the anammox bacteria, e.g. *Ca*. Scalindua, and Beggiatoaceae bacteria, which were present in the host sediments at both locations.

At Station 23002, the most abundant and enriched ASV in the filamentous Beggiatoaceae sample was the sulfur-oxidizer Campylobacterota, *Sulfurovum*. ASVs of *Sulfurovum* were also very abundant in these sediments, as well as the non-filamentous Beggiatoaceae sample from this location. The genus is usually thought to occur as small rods, but large filamentous strains do exist [100, 101]. Thus, it is possible a filamentous strain of *Sulfurovum* were collected alongside that of the Beggiatoaceae but *Sulfurovum* does not possess intracellular sulfur globules, a distinguishing characteristic of members of the Beggiatoaceae. Other ASVs noticeably-enriched in the Station 23002 filamentous Beggiatoaceae sample were uncharacterized gammaproteobacteria from the orders CH2b56 and eub62A3, but neither were enriched in the Station 20020 samples. In filamentous Beggiatoaceae samples from Station 20020, we identified several bacteria unclassified at the phylum level that may have an association with these Beggiatoaceae including the second most abundant ASV in the sample, as well as a nanoarchaeon ASV. None of the ASVs that appeared to be enriched in samples from Station 20020 were enriched in filamentous Beggiatoaceae from station 23002. We tried to further explore the identity of the second most abundant ASV in the 23002 sample by blasting it against the NCBI non-redundant nucleotide collection, but the closest match had only an 84.52% identity. The most abundant ASV in all three station 20020 filamentous Beggiatoaceae samples was likely a plastid from the benthic foraminifera *Virgulinella fragilis* (100% JN207219). Of the larger two sample DNA libraries the foraminifera plastid exceeded 15,000 ASVs vs 0–4500 in the six host sediment samples. Protists have been found inside the sheath of cold seep *Ca*. Marithioploca spp. from Monterey Bay [102]. And it was postulated that the enclosed sheath environment, where the concentration of hydrogen sulfide is likely lower than ambient conditions, may serve as a refuge for hypoxia tolerant protists like *Virgulinella fragilis*.

### ASVs uniquely associated with non-filamentous Beggiatoaceae

**Fig 6:**
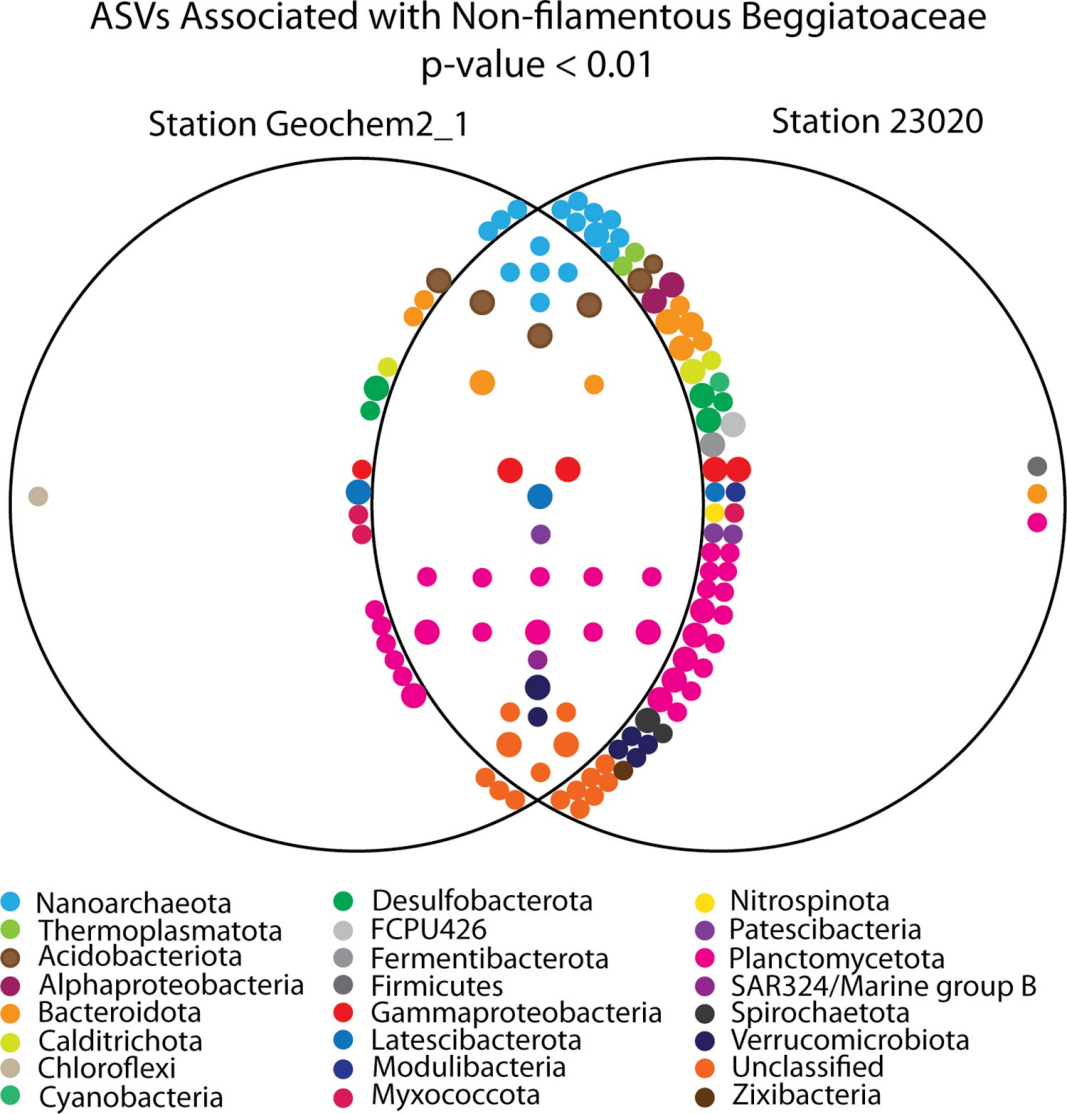
Modified Venn diagram of 123 ASVs detected by DESeq2 as having a statistically significant association with non-filamentous Beggiatoaceae hosts from Marine Station Geochem2_1 and Marine Station 23020. ASVs represented in both circles were found to have a significant association with the hosts from both stations. ASVs neighboring the overlapping circles, we determined likely also were significantly enriched the other station but a single low representative inhibited detection via DESeq2. ASVs represented by large circles appeared to also be enriched in the sample from shallow station 23002.

We collected six samples non-filamentous Beggiatoaceae from Station 23020, three samples from GeoChem2_1 and one sample of from the shallow Station 23002. Thus, we used DESeq2 to identify ASVs significantly associated with the non-filamentous Beggiatoaceae from Stations 23020 and GeoChem2_1, S1 Table, but not Station 23002. DESeq2 detected 55 ASVs in Station GeoChem2_1 and 100 ASVs in Station 23020 samples with significance association with host non-filamentous Beggiatoaceae, presented in a modified Venn Diagram, Fig 6. Thirty-two of those ASVs were found to be significantly associated with non-filamentous Beggiatoaceae from both stations. Closer examination of the raw data for the other 91 ASVs indicated that there were other likely significant associations not detected by DESeq2 because of low abundance in one non-filamentous Beggiatoaceae replicate or low-level abundance in host sediment from which the non-filamentous Beggiatoaceae was removed. We found that all but three ASVs from the collective 123 unique ASVs were statistically significantly enriched in the non-filamentous Beggiatoaceae samples between the two stations. We also queried the raw data of shallow Station 23002 for the presence of the 123 enriched ASVs at the other two locations and then compared their abundances verses the host sediment and sediment cores. Thirty-five positive results are highlighted in Fig 6 by larger circles representing ASVs.

The most abundant phyla with significant association with the non-filamentous Beggiatoaceae was the Planctomycetota, the phylum containing anammox bacteria. But none of the 33 Planctomycetota ASVs were of clades for which anammox is known to occur. Instead, 10 ASVs were unclassified at the class level, 11 were from the OM190, 6 from the Phycisphaerales, and 5 were from the Pla4 lineage. One of the unclassified ASVs was also very enriched (300 vs. <20) in the sample from the shallower site, Station 23002. One low-abundance ASV found in Station 23020 samples from the Pla3 lineage was not detected in either of the other two station samples. Members of both the OM190 clade and the Phycisphaerales have been found to be attached to macroalgae and are thought to be organoheterotrophs that degrade algal sulfated polysaccharides [103]. Members of the Planctomycetota from the Benguela Upwelling OMZ have been shown to possess a large number of sulfatases [104]. We hypothesize that certain Planctomycetota may attach to *Ca*. Thiomargarita’s sheath to degrade sulfated polysaccharides and other sheath constituents.

There were other clades of chemoorganotrophs/fermenters associated with the non-filamentous Beggiatoaceae samples. Members of the Bacteriodota are known to degrade sulfated polysaccharides [105] and have been previously observed attached to the sheath of filamentous *Sulfurovom* [101]. Of the ten ASVs within the Bacteriodota that were found to be associated with the non-filamentous samples, three were within the Bacteroidetes BD2-2 family, two were within the Flavobacteriaceae, one in the Lentimicrobiaceae, two in the Melioribacteraceae, and two were within unclassified clades of the Bacteroidales. Other chemoorganotrophs/fermenters included two ASVs of Kiritimatiellae (Verrucomicrobiota) [106]; three Latescibacterota [107]; three Calditrichota [108]; three Myxococcota [109]; six Acidobacteria (predominantly subgroups 22 and 23) [110], two Spirochaetota [111] and one ASV each of Lentisphaeria (Verrucomicrobiota) [112], candidate phylum FCPU426 [113], Fermentibacterota [114], Ardenticatenales Chloroflexi [115], Gastranaerophilales cyanobacterium (aka. Melainabacteria) [116], unclassified Firmicutes [117], Modulibacteria [118] and Zixibacteria [119]. Most of these clades are poorly studied, but there have been many observations of morphologically-distinct members of the Spirochaetota in association with the Beggiatoaceae [120–122]. Many of these clades likely respire nitrogenous species and/or generate H_2_ and/or low molecular weight organic acids as byproducts of fermentation. *Ca*. Thiomargarita spp. have the capacity to metabolize both acetate and hydrogen [20]. Thus in some cases, chemoorganotrophs/fermenters may be beneficial to their Beggiatoaceae host [101]. Additionally, syntrophic relationships likely exist concerning the exchange of metabolic cofactors such as B-vitamins [123]. Some of these ASVs were also noticeably enriched in the sample from station 23002, e.g., a Bacteroidetes BD2-2 (1400 vs. < 20); and three Subgroup 23 Acidobacteriota (450 vs. 0)(2x 300 vs.<10). Also enriched in the non-filamentous Beggiatoaceae sample from Station 23002 (but not the other two stations) was an ASV of an unclassified Planctomycetota (300 vs. <20); a Maritimimonas flavobacterium (300 vs. 0); a Turicella of Actinobacteriota (300 vs. 0); a Spirochaeta_2 (300 vs. <20); and an Acinetobacter (250 < 0).

In addition to free-living chemoorganotrophs and fermenters, a number of ASVs may depend on the host *Ca*. Thiomargarita spp. for their nutrition either in a syntrophic or predatory relationship. Both stations had an ASV of a Patescibacteria (*Ca*. Komeilibacteria family) which have reduced genomes [124] and are thought to be dependent on chemolithoautrophic hosts for intermediates [125]. Nanoarchaea was one of the most abundant phyla in at all of the marine stations, 99.92% of which are within the class Woesearchaeales. Like the Patescibacteria, the Woesearchaeota have reduced genomes and are thought to be dependent on hosts for metabolic intermediates [126]. Liu et al, 2018 proposed that methanogens would be an ideal host because the Woesearchaeota are capable of acetate fermentation and hydrogen production which are key to many methanogenesis reactions. But likewise, acetate and hydrogen can also be utilized by members of the Beggiatoaceae. DESeq2 found 15 Woesearchaeota ASVs that were significantly associated with non-filamentous Beggiatoaceae. Yet for such an exchange of nutrients to occur, there would likely need to be direct cell-to-cell contact between the epibiont and the host, which would require the patescibacterium and the Woesearchaeota to have the capacity to penetrate the host’s sheath. We have observed considerable variability in the thickness of *Ca*. Thiomargarita spp. sheath with barrel shaped *Ca*. T. nelsonii having the thickest sheaths [2] and thus suggest these relationships may exist in a subset of non-filamentous Beggiatoaceae morphotypes. ASVs of these two phyla were not enriched in the sample from Station 23002.

Syntrophic interactions have been demonstrated between sulfate-reducing bacteria and sulfur-oxidizing bacteria [127–129] and putative sulfate-reducing bacteria have been previously observed on the exteriors of *Thiomargarita* [59]. As anticipated based on these previous observations, putative sulfate reducers were significantly associated with the non-filamentous Beggiatoaceae of the phyla Desulfobacterota (formerly known as the Deltaproteobacteria) and Thermoplasmatota. Sulfate-reducing Desulfobacterota have been previously identified as having co-associations with members of the Beggiatoaceae [55, 98]. Some ASVs associated with non-filamentous Beggiatoaceae were in relatively low abundances, i.e. at Station 23020 an ASV of Desulfocaspa and an unclassified Desulfobulbales and at Station GeoChem2_1, an ASV of Desulfuromusa. Additionally, in the sample from the shallow Station 23002, a Desulfobacterales (300 vs. <20) and a Desulfobulbales (250 vs. 0) appeared to be enriched. Two strains of Desulfosarcinaceae were very abundant in samples from both Station 23020 and Geochem2_1. One strain was also quite abundant in cores (ASV 3) from all four stations but appeared to be enriched in non-filamentous Beggiatoaceae samples from Station 23020. The other strain of Desulfosarcinaceae was only found in non-filamentous Beggiatoaceae samples and not in the sediments from all three stations where the non-filamentous Beggiatoaceae were collected. One replicate from Station 23020 did not have this ASV, and thus it wasn’t found by DESeq2 to be statistically significant in its association with non-filamentous Beggiatoaceae at this location. The Desulfosarcinaceae is a metabolically diverse clade but all representatives are sulfate-reducing bacteria. Many strains have been found to be key in syntrophic interactions such as AOM [130], anaerobic ethane oxidation [131] and butyrate-dependent syntrophy [132]. They have also been found to be intracellular symbionts of foraminifera [133] and ciliates [134] in which they provide their fermentative host with amino acids. The potential for sulfate-reduction in ASVs of Thermoplasmatota is suggested by the habitat and unclassified status of the two ASVs that were significant in Station 23020 samples. This class of archaea has been reclassified [83] to include a number of archaeal groups including methanogens (Methanomassiliicoccales), photoheterotrophs *Ca*. Poseidoniales (formerly Marine Group II), and other heterotrophs (Marine Group III and DHVEG-6 [135]. But recently, unclassified members of Thermoplasmatota have been shown to have the genetic potential for sulfate-reduction, hydrogen oxidation and denitrification as well as heterotrophy and fermentation [136].

Surprisingly, putative strains of sulfur oxidizers were significantly associated with non-filamentous Beggiatoaceae. The sulfur-oxidizers were three ASVs of Omnitrophia (Verrucomicrobiota), members of which are thought to oxidize sulfur with iron as an electron acceptor [137], one low abundance ASV of the SAR 324 clade [138], two unclassified Gammaproteobacteria, two Thiotrichaceae Gammaproteobacteria [139] and one highly enriched ASV of an unknown family of the Gammaproteobacteria Incertae Sedis [140], which was also very abundant in the Station 23002 sample (1200 vs. <10). In addition, two ASVs of the Alphaproteobacteria clades Rhizobiales [141] and Rhodobacterales could represent organoheterotrophic sulfur-oxidizers [142]. The ASV of the Rhizobiales was also noticeably enriched in the sample from Station 23002 (200 vs. <10) as well as an unclassified gammaproteobacterium (240 vs. 0). Many organoheterotrophic sulfur-oxidizers, particularly those within the Alphaproteobacteria are very adept at degrading organosulfur compounds, which may be beneficial to co-associated inorganic sulfur oxidizers [143]. The primary sulfur-oxidizers that were enriched in the shallower site, Station 23002, were from the phylum Campylobacterota. Indeed, the most abundant and highly enriched ASV in the non-filamentous Beggiatoaceae sample was a strain of the Campylobacterota *Sulfurimonas* [144] (>16,000 vs. less than 500 in sediment samples). The *Sulfurovum* ASV that was enriched in the filamentous Beggiatoaceae sample from shallow Station 23002 was also abundant but not necessarily enriched in the non-filamentous Beggiatoaceae sample from the same station.

Both the filamentous and non-filamentous Beggiatoaceae were strongly associated with sulfur-oxidizing Campylobacterota at the shallow Station 23002, but not at other stations where the Beggiatoaceae were more abundant. Several studies have proposed a difference in niche preferences between sulfur-oxidizers within the Campylobacterota vs. the Gammaproteobacteria, including members of the Beggiatoaceae. In general, Campylobacterota dominate in sediments with higher concentrations of sulfide potentially due to greater sensitivity to O_2_ [50, 145–148]. Often these sulfide-rich sediments contain the intermediates of sulfur respiration and disproportionation such as S^0^. Some Campylobacterota, can incompletely oxidize sulfide to S^0^ which is then excreted [149, 150] while others, such as strains of *Sulfurimonas*/*Sulfurovum* are thought to be able to oxidize extracellular S^0^, including cyclooctasulfur (S_8_), a trait unique to the Campylobacterota [151, 152]. Some strains of Campylobacterota from hydrothermal vent systems reduce extracellular S_8_ and/or other S intermediates when H_2_ is available [153–155], much like respiration of internal sulfur globules by members of the Beggiatoaceae. Whether this occurs in other settings is unknown. One Campylobacterota sulfur-reducer at hydrothermal vents is *Thiofractor thiocaminus*, the sole isolate from this genus [154]. An ASV of *Thiofractor* was the second most abundant ASV in the non-filamentous Beggiatoaceae sample from Station 23002 (8,000 vs. >100 in sediment samples). Whether these Campylobacterota are functioning as sulfur-oxidizers or sulfate reducers in this setting remains to be determined. We propose that attached sulfur-oxidizing Campylobacterota may be key to Beggiatoaceae bacteria inhabiting these highly sulfidic sediments by reducing the concentration of sulfide in the diffusion boundary layer surrounding the Beggiatoaceae cells [156]. Hydrogen sulfide can be deleterious to cells when one electron is oxidized producing certain sulfur intermediates [157, 158]. While both clades must have a variety of protective responses, these Campylobacterota may have evolved more efficient/effective adaptations to maintain cellular homeostasis to include producing extracellular products of the oxidation of sulfide (vs. the more metabolically expensive mechanism of funneling S_red_ into intracellular sulfur globules). The production of extracellular sulfur intermediates by the Campylobacterota may also be coupled to the metabolism of attached strains that can disproportionate sulfur, such as members of the Desulfobacterales [159, 160] creating microenvironments of robust sulfur cycling.

We anticipated that a number of enriched ASVs would be ammonium and nitrite oxidizing bacteria, because inorganic nitrogen cycling could help sustain viability between occasional exposure to O_2_ [161]. Instead, many of the ASVs associated with the non-filamentous Beggiatoaceae were more likely to be denitrifiers and ammonifiers. If these bacteria respire nitrate, they would be competing with their Beggiatoaceae hosts for that electron acceptor. As in the case of the filamentous Beggiatoaceae, anammox bacteria were present in the host sediments containing these Beggiatoaceae but they were not enriched in the Beggiatoaceae samples. These sediments also contained ASVs from an uncharacterized clade of Nitrosprirota. Members of this phylum are known to carry out complete aerobic nitrification of ammonia to nitrate [162, 163], but these ASVs were also not enriched in the non-filamentous Beggiatoaceae samples. Neither were members of the Nitrosococcales that were in low abundances in these sediments. DESeq2 found only one statistically significant association between a potential microoxic nitrifier of nitrite to nitrate within an unclassified clade distal to known nitrite nitrifiers within the Nitrospinota [164, 165]. This ASV was of low abundance but was clearly enriched in samples from Station 23020, and to a lesser extent, samples from Station GeoChem2_1.

We were unable to find any indication of enrichment of autotrophic ammonium or nitrite oxidizers in the sample from the shallow site, Station 23002. There was some indication of enrichment of clades known for heterotrophic nitrification, like members of the Pseudomonadales and Comamonadaceae, which were not detected in the host sediment. This metabolic capability has been poorly studied outside of wastewater treatment, e.g., [166, 167], and the clades capable of heterotrophic nitrification, particularly in marine settings, have not been well explored. In some cases, they are mixotrophs that possess ammonia monooxygenases and hydroxylamine oxidoreductases homologous to those of autotrophic nitrifiers, thus they likely derive energy from them [168]. But in other cases, the enzymes appear to be of a different origin [167, 169–172]. These heterotrophic nitrifiers are also often capable of aerobic denitrification and are sometimes sulfur-oxidizing bacteria [173]. This metabolism is thought to conserve O_2_ for respiration under low O_2_ conditions by reducing the electron flow to O_2_ [174]. Some aerobic ammonia-oxidizers and nitrifiers can couple these processes to denitrification [167, 175] under anaerobic conditions, which sometimes results in the release of NO [176, 177] and N_2_O [178, 179] or they denitrify using other electron donors such as H_2_ [180].

Another potential metabolism that would involve cycling of inorganic nitrogenous compounds anaerobically is sulfammox, (Eq 1) [181, 182].


$$8NH_{4}{}^{+}+3SO_{4}{}^{2‐}\to 4N_{2}+3HS^{‐}+12H_{2}O+5H^{+}\;\Delta G_{0}\prime =‐17.6\;KJ\;mol^{‐1}\;(pH\;7)$$ Eq 1

Like heterotrophic nitrification, this process has primarily been studied in wastewater treatment [183] but has recently been detected in marine settings [80, 184]. In many natural settings, sulfammox is not thermodynamically favorable at ambient conditions, often requiring either high ammonium or the removal to the intermediate nitrite. Autotrophic sulfide-oxidizers capable of denitrification have long been implemented in facilitating sulfammox by both removing nitrite but also reduced sulfur species, e.g., [185–189]. Currently, the clade(s) responsible for sulfammox are unknown, but traditional anammox bacteria and other Planctomycetes clades [186–190] including the Phycisphaeraceae [184], as well as members of the Verrucomicrobiales [187–189] have been suggested to play a role in the process. One isolated strain of *Bacillus* sp. was thought to perform sulfammox [191] and recently, a Betaproteobacterium, *Ralstonia* sp., has been isolated that performs this reaction, but this *Ralstonia* sp. prefers thiosulfate over sulfate as an electron acceptor [192]. Currently, the metabolic pathways that support these sulfammox processes are undetermined, but in many cases, nitrite is commonly detected as an intermediate, e.g. [192]. The production of nitrite along with the production of reduced sulfur species would be highly beneficial to members of the Beggiatoaceae. Furthermore, Beggiatoaceae performing DNRA and sulfide-oxidation would be beneficial hosts for sulfammox bacteria that require both sulfate or thiosulfate and ammonium.

Lastly, there were a total of 16 bacterial ASVs that were unclassified at the phylum level, but which were detected by DESeq2 as having a significant association with non-filamentous Beggiatoaceae samples. We attempted to determine their last known common ancestor, S5 Fig. Six of these ASVs were too distant from sequences within the Silva database to infer the ancestral origin. The Acidobacteria appear to be the last common ancestor of five ASVs and the Planctomycetes of two ASVs. Three other ASVs had the common ancestral phyla of the Spirochaetota, Bdellovibrionata, and the Myxococcota. Five ASVs were statically significant in samples from both Station 23020 and Geochem2_1 and one of which was highly enriched in the sample from Station 23002 (1300 vs. <10). The sample from Station 23002 also had an unclassified bacterial ASV that was one of the most abundant ASVs in the sample and was highly enriched comparison to the host sediments (2700 vs. < 100). We postulate that many of these ASVs may represent strains that have evolved to be specifically host-adapted to members of the non-filamentous Beggiatoaceae as either syntrophs or parasites.

## Conclusion

The objectives of this study were two-fold: 1) to learn more about the environmental parameters of the ecotypes of motile (filamentous) vs. non-motile (non-filamentous) members of the Beggiatoaceae; 2) to determine if there may be select microbes preferentially attached or associated with to these of the Beggiatoaceae. Our sediment core pore water geochemistry profiles revealed that both filamentous and non-filamentous Beggiatoaceae can be found under a broad range of oxygen and sulfide concentrations. Under high concentrations of sulfide we observed very abundant host associated members of the Campylobacterota, which are known to prefer regimes with high sulfide to oxygen ratios. We hypothesize that they may reduce the concentration of sulfide around the host Beggiatoaceae promoting an environmental setting that is more amenable to the Beggiatoaceae that are less tolerant of extremely sulfidic conditions. In these sulfide-rich sediments, we also found enriched ASVs of the *Desulfonema* and foraminifera in the filamentous Beggiatoaceae sample, as previously reported to be associated with filamentous Beggiatoaceae at other environments. These associations were not found in the deeper water settings, where sulfide concentrations are lower in the sediments. However, in deeper settings, another previously reported Beggiatoaceae host association was found, that of the aerobic ammonium oxidizer *Ca*. Nitrosopumilus. In general, we found fewer specific associations between the filamentous Beggiatoaceae and their epibiont community than we found with the non-filamentous Beggiatoaceae, even after taking into account DNA library size biases. But previously unreported associations appear to exist, including those with strains that are unclassifiable at the phylum level.

Outside of the sulfidic shallow sediments, the non-filamentous Beggiatoaceae were predominately found in the heart of the mud belt sediments, which are prone to gas ebullition. We found 123 ASVs to be enriched in Beggiatoaceae from these sediments many of which are likely capable of syntrophic metabolisms supplying their hosts with organic acids and hydrogen. Others may compete with their hosts for nutrients such as nitrate and sulfide or have unknown metabolic potential. We expected to find an association between anammox bacteria and non-filamentous Beggiatoaceae but found no such relationship. Instead, we found a nitrite oxidizer enriched in the host sample relative to the sediment, but in low abundance. Since inorganic nitrogen is key to respiration in these non-motile strains and both energy and carbon sources are likely provided by epibionts, we propose that other less well-studied metabolic processes such as heterotrophic nitrification and sulfammox may be supplying these Beggiatoaceae with nitrate and/or nitrite.

A significant limitation of our study was that the Beggiatoaceae were pooled based on morphology thus combining different genera and species, so exact host association remains elusive. Additionally, phylogenetic identification of taxa using amplicon sequencing is not a perfect predictor of metabolic potential, although many of the ASVs reported on here represent clades with distinctive metabolic characteristics. Whole genome amplification of individual Beggiatoaceae chains and filaments for metagenomic analyses will further elucidate these relationships. Niche preference by individual species could also be improved by analyzing the host sediment particle size and composition, including the percent of organic matter, as well as collecting additional porewater chemistry including other potential microbial substrates such as methane. While the results of this study have left us with more questions than answers, they reveal that the Beggiatoaceae are more than bacteria: they are hosts of their own microbiomes.

## Supporting information

S1 Fig Water column dissolved oxygen profiles off Namibia collected as part of the regional graduate networks in oceanography survey April 2017. A. Along the -20 latitude, B. Along the -23 latitude, C. At Marine Station Geochem2_1 April 2017.
(TIF)

S2 Fig Hierarchical cluster analysis via euclidean distance of the 16S rRNA community composition of samples collected at four marine stations: 23002, 20020, 23020 and Geochem2_1 and various depths between 0–5 cm. (TIF)

S3 Fig Mean species diversity via the Chao1 (sampling depth) and Shannon (evenness of ASVs) indices of the 16S rRNA communities of samples collected from four marine stations: 23002, 20020, 23020 and Geochem2_1 and at various depths between 0–5 cm. (TIF)

S4 Fig Rarefaction plots of 16S rRNA communities of samples collected from four marine stations: 23002, 20020, 23020 and Geochem2_1 and at various depths between 0–5 cm demonstrate that some samples were of lower sequencing depth than most samples. The plots demonstrate that some samples had a small library size which failed to adequate capture the entire community, which likely resulted in under detection via DESeq2 of some significant associations between host Beggiatoaceae and their attached epibionts.
(TIF)

S5 Fig Neighbor joining 16S rRNA gene phylogenic distance tree of ASVs with statistically significant association with non-filamentous Beggiatoaceae along with top NCBI blastn match. The 16S rRNA gene sequences were classified against the Silva database using the SINA aligner 1.2.1.1 with a 70% identity cutoff. Red stars indicated that the ASV was statistically significant for both stations 23020 and Geochem2_1 samples. Blue stars indicate that the ASV was likely associated with the non-filamentous LSB sample from Marine Station 23002.
(TIF)

S1 Text Discussion of geochemical profiles of Marine Station 23002 sediments collected April 2017. (DOCX)

S1 Table DeSeQ2 identified significant ASVs in Beggiatoaceae samples vs. their host sediments for Marine Station 20020, Marine Station 23020 and Marine Station GeoChem2_1. (XLSX)

## References

[1] Jørgensen BB, Findlay AJ, Pellerin A. The Biogeochemical Sulfur Cycle of Marine Sediments. Front Microbiol. 2019;10:849. Epub 2019/05/21. doi: 10.3389/fmicb.2019.00849 ; PubMed Central PMCID: PMC6492693. 31105660 PMC6492693

[2] Salman V, Bailey JV, Teske A. Phylogenetic and morphologic complexity of giant sulphur bacteria. Antonie Leeuwenhoek. 2013;104(2):169–86. Epub 2013/06/25. doi: 10.1007/s10482-013-9952-y . 23793621

[3] Schulz HN, Brinkhoff T, Ferdelman TG, Marine MH, Teske A, Jørgensen BB. Dense populations of a giant sulfur bacterium in Namibian shelf sediments. Science. 1999;284(5413):493–5. Epub 1999/04/16. doi: 10.1126/science.284.5413.493 . 10205058

[4] Parks DH, Chuvochina M, Chaumeil PA, Rinke C, Mussig AJ, Hugenholtz P. A complete domain-to-species taxonomy for Bacteria and Archaea. Nat Biotechnol. 2020;38(9):1079–86. Epub 2020/04/29. doi: 10.1038/s41587-020-0501-8 . 32341564

[5] Parks DH, Chuvochina M, Waite DW, Rinke C, Skarshewski A, Chaumeil PA, et al. A standardized bacterial taxonomy based on genome phylogeny substantially revises the tree of life. Nat Biotechnol. 2018;36(10):996–1004. Epub 2018/08/28. doi: 10.1038/nbt.4229 . 30148503

[6] Teske A, Salman V. The Family Beggiatoaceae. The Prokaryotes Berlin Heidelberg: Springer-Verlag 2014. p. 93–134.

[7] Prange A, Chauvistre R, Modrow H, Hormes a J, Truper H, Dahl C. Quantitative speciation of sulfur in bacterial sulfur globules: X-ray absorption spectroscopy reveals at least three different species of sulfur. Microbiology. 2002;148(1):267–76. doi: 10.1099/00221287-148-1-267 11782519

[8] Pasteris JD, Freeman JJ, Goffredi SK, Buck KR. Raman spectroscopic and laser scanning confocal microscopic analysis of sulfur in living sulfur-precipitating marine bacteria. Chemical Geology. 2001;180(1–4):3–18.

[9] Berg JS, Schwedt A, Kreutzmann A-C, Kuypers MMM, Milucka J. Polysulfides as intermediates in the oxidation of sulfide to sulfate by *Beggiatoa* spp. Appl Environ Microb. 2014;80(2):629–36. doi: 10.1128/AEM.02852-13 24212585 PMC3911116

[10] Nelson DC, Jørgensen BB, Revsbech NP. Growth pattern and yield of a chemoautotrophic *Beggiatoa* sp. in oxygen-sulfide microgradients. Appl Environ Microb. 1986;52(2):225–33. Epub 1986/08/01. doi: 10.1128/aem.52.2.225-233.1986 ; PubMed Central PMCID: PMC203507. 16347121 PMC203507

[11] Schmidt T, Arieli B, Cohen Y, Padan E, Strohl W. Sulfur metabolism in *Beggiatoa alba*. J Bacteriol. 1987;169(12):5466–72. Epub 1987/12/01. doi: 10.1128/jb.169.12.5466-5472.1987 PubMed Central PMCID: PMC213973. 3316186 PMC213973

[12] Schwedt A, Kreutzmann AC, Polerecky L, Schulz-Vogt HN. Sulfur respiration in a marine chemolithoautotrophic *Beggiatoa* strain. Front Microbiol. 2011;2:276. Epub 2012/02/01. doi: 10.3389/fmicb.2011.00276 ; PubMed Central PMCID: PMC3253548. 22291687 PMC3253548

[13] Schmidt TM, Vinci VA, Strohl WR. Protein synthesis by *Beggiatoa alba* B18LD in the presence and absence of sulfide. Arch Micro. 1986;144(2):158–62.

[14] Kreutzmann AC, Schulz-Vogt HN. Oxidation of molecular hydrogen by a chemolithoautotrophic B*eggiatoa* strain. Appl Environ Microbiol. 2016;82(8):2527–36. Epub 2016/02/21. doi: 10.1128/AEM.03818-15 ; PubMed Central PMCID: PMC4959497. 26896131 PMC4959497

[15] Hagen KD, Nelson D. Organic carbon utilization by obligately and facultatively autotrophic *Beggiatoa* strains in homogeneous and gradient cultures. Appl Environ Microb. 1996;62(3):947–53. doi: 10.1128/aem.62.3.947-953.1996 16535281 PMC1388806

[16] MacGregor BJ, Biddle JF, Harbort C, Matthysse AG, Teske A. Sulfide oxidation, nitrate respiration, carbon acquisition, and electron transport pathways suggested by the draft genome of a single orange Guaymas Basin *Beggiatoa* (*Cand.* Maribeggiatoa) sp. filament. Mar Genomics. 2013;11:53–65. Epub 2013/09/10. doi: 10.1016/j.margen.2013.08.001 . 24012537

[17] Flood BE, Fliss P, Jones DS, Dick GJ, Jain S, Kaster A-K, et al. Single-cell (meta-)genomics of a dimorphic *Candidatus* Thiomargarita nelsonii reveals genomic plasticity. Front Microbiol. 2016;7:603. Epub 2016/05/21. doi: 10.3389/fmicb.2016.00603 ; PubMed Central PMCID: PMC4853749. 27199933 PMC4853749

[18] Nelson DC, Castenholz RW. Organic nutrition of *Beggiatoa* sp. J Bacteriol. 1981;147(1):236–47. Epub 1981/07/01. doi: 10.1128/jb.147.1.236-247.1981 ; PubMed Central PMCID: PMC216030. 7240094 PMC216030

[19] Maier S, Gallardo VA. Nutritional characteristics of two marine thioplocas determined by autoradiography. Arch Micro. 1984;139(2–3):218–20.

[20] Bailey JV, Flood BE, Ricci E, Delherbe N. Imaging of cellular oxidoreductase activity suggests mixotrophic metabolisms in *Thiomargarita* spp. mBio. 2017;8(6):e01263–17. Epub 2017/11/09. doi: 10.1128/mBio.01263-17 ; PubMed Central PMCID: PMC5676036. 29114021 PMC5676036

[21] Nelson DC, Castenholz RW. Use of reduced sulfur compounds by *Beggiatoa* sp. J Bacteriol. 1981;147(1):140–54. doi: 10.1128/jb.147.1.140-154.1981 7240091 PMC216018

[22] Grabovich MY, Patritskaya VY, Muntyan MS, Dubinina GA. Lithoautotrophic growth of the freshwater strain *Beggiatoa* D-402 and energy conservation in a homogeneous culture under microoxic conditions. FEMS Microbiol Lett. 2001;204(2):341–5. Epub 2001/12/04. doi: 10.1111/j.1574-6968.2001.tb10908.x . 11731146

[23] Rudenko TS, Tarlachkov SV, Shatskiy ND, Grabovich MY. Comparative genomics of *Beggiatoa leptomitoformis* strains D-401 and D-402(T) with contrasting physiology but extremely high level of genomic identity. Microorganisms. 2020;8(6). Epub 2020/06/25. doi: 10.3390/microorganisms8060928 ; PubMed Central PMCID: PMC7356093. 32575621 PMC7356093

[24] Jewell T, Huston SL, Nelson DC. Methylotrophy in freshwater *Beggiatoa alba* strains. Appl Environ Microb. 2008;74(17):5575–8. Epub 2008/07/16. doi: 10.1128/AEM.00379-08 ; PubMed Central PMCID: PMC2546629. 18621874 PMC2546629

[25] Orlova MV, Tarlachkov SV, Kulinchenko EI, Dubinina GA, Tutukina MN, Grabovich MY. Genomics and biochemistry of metabolic pathways for the c1 compounds utilization in colorless sulfur bacterium *Beggiatoa leptomitoformis* D-402. Indian J Microbiol. 2018;58(4):415–22. Epub 2018/09/29. doi: 10.1007/s12088-018-0737-x ; PubMed Central PMCID: PMC6141403. 30262951 PMC6141403

[26] Møller MM, Nielsen LP, Jørgensen BB. Oxygen responses and mat formation by *Beggiatoa* spp. Appl Environ Microb. 1985;50(2):373–82. Epub 1985/08/01. doi: 10.1128/aem.50.2.373-382.1985 ; PubMed Central PMCID: PMC238630. 16346857 PMC238630

[27] Nelson DC, Revsbech NP, Jørgensen BB. Microoxic-anoxic niche of *Beggiatoa* spp.: microelectrode survey of marine and freshwater strains. Appl Environ Microb. 1986;52(1):161–8. Epub 1986/07/01. doi: 10.1128/aem.52.1.161-168.1986 ; PubMed Central PMCID: PMC203429. 16347103 PMC203429

[28] Schulz HN, de Beer D. Uptake rates of oxygen and sulfide measured with individual *Thiomargarita namibiensis* cells by using microelectrodes. Appl Environ Microb. 2002;68(11):5746–9. doi: 10.1128/AEM.68.11.5746-5749.2002 12406774 PMC129903

[29] Schutte CA, Teske A, MacGregor BJ, Salman-Carvalho V, Lavik G, Hach P, et al. Filamentous giant Beggiatoaceae from the Guaymas Basin are capable of both denitrification and dissimilatory nitrate reduction to ammonium. Appl Environ Microb. 2018;84(15). doi: 10.1128/AEM.02860-17 29802192 PMC6052272

[30] McHatton SC, Barry JP, Jannasch HW, Nelson DC. High nitrate concentrations in vacuolate, autotrophic marine *Beggiatoa* spp. Appl Environ Microb. 1996;62(3):954–8. Epub 1996/03/01. doi: 10.1128/aem.62.3.954-958.1996 ; PubMed Central PMCID: PMC1388807. 16535282 PMC1388807

[31] Fossing H, Gallardo VA, Jørgensen BB, Hüttel M, Nielsen LP, Schulz H, et al. Concentration and transport of nitrate by the mat-forming sulphur bacterium *Thioploca*. Nature. 1995;374(6524):713–5.

[32] Huettel M, Forster S, Kloser S, Fossing H. Vertical migration in the sediment-dwelling sulfur bacteria *Thioploca* spp. in overcoming diffusion limitations. Appl Environ Microb. 1996;62(6):1863–72. Epub 1996/06/01. doi: 10.1128/aem.62.6.1863-1872.1996 ; PubMed Central PMCID: PMC1388866. 16535328 PMC1388866

[33] Mußmann M, Schulz HN, Strotmann B, Kjær T, Nielsen LP, Rosselló‐Mora RA, et al. Phylogeny and distribution of nitrate‐storing *Beggiatoa* spp. in coastal marine sediments. Environ Microbiol. 2003;5(6):523–33. Epub 2003/05/21. doi: 10.1046/j.1462-2920.2003.00440.x . 12755720

[34] Beutler M, Milucka J, Hinck S, Schreiber F, Brock J, Mußmann M, et al. Vacuolar respiration of nitrate coupled to energy conservation in filamentous Beggiatoaceae. Environ Microbiol. 2012;14(11):2911–9. Epub 2012/08/29. doi: 10.1111/j.1462-2920.2012.02851.x . 22925165

[35] Buckley A, MacGregor B, Teske A. Identification, expression and activity of candidate nitrite reductases from orange Beggiatoaceae, Guaymas Basin. Front Microbiol. 2019;10:644. Epub 2019/04/16. doi: 10.3389/fmicb.2019.00644 ; PubMed Central PMCID: PMC6449678. 30984153 PMC6449678

[36] Nelson DC, Waterbury JB, Jannasch HW. Nitrogen fixation and nitrate utilization by marine and freshwater *Beggiatoa*. Arch Micro. 1982;133(3):172–7.

[37] Gureeva MV, Belousova EV, Dubinina GA, Novikov AA, Kopitsyn DS, Grabovich MY. *Thioflexithrix psekupsensis* gen. nov., sp. nov., a filamentous gliding sulfur bacterium from the family Beggiatoaceae. Int J Syst Evol Microbiol. 2019;69(3):798–804. Epub 2019/01/19. doi: 10.1099/ijsem.0.003240 . 30657444

[38] Nelson DC, Castenholz RW. Light responses of *Beggiatoa*. Arch Micro. 1982;131(2):146–55.

[39] Bremner J, Willis J. Mineralogy and geochemistry of the clay fraction of sediments from the Namibian continental margin and the adjacent hinterland. Marine Geology. 1993;115(1–2):85–116.

[40] Louw DC, van der Plas AK, Mohrholz V, Wasmund N, Junker T, Eggert A. Seasonal and interannual phytoplankton dynamics and forcing mechanisms in the Northern Benguela upwelling system. J Mar Syst. 2016;157:124–34. doi: 10.1016/j.jmarsys.2016.01.009

[41] Flynn RF, Granger J, Veitch JA, Siedlecki S, Burger JM, Pillay K, et al. On‐shelf nutrient trapping enhances the fertility of the Southern Benguela upwelling system. J Geophys Res Oceans. 2020;125(6). doi: 10.1029/2019jc015948

[42] Emeis K, Eggert A, Flohr A, Lahajnar N, Nausch G, Neumann A, et al. Biogeochemical processes and turnover rates in the Northern Benguela Upwelling System. J Mar Syst. 2018;188:63–80. doi: 10.1016/j.jmarsys.2017.10.001

[43] Ohde T, Dadou I. Seasonal and annual variability of coastal sulphur plumes in the northern Benguela upwelling system. PLoS ONE. 2018;13(2):e0192140. Epub 2018/02/09. doi: 10.1371/journal.pone.0192140 ; PubMed Central PMCID: PMC5805238. 29420587 PMC5805238

[44] Brüchert V, Currie B, Peard KR, Lass U, Endler R, Dübecke A, et al. Biogeochemical and physical control on shelf anoxia and water column hydrogen sulphide in the Benguela coastal upwelling system off Namibia. Past and present water column anoxia: Springer; 2006. p. 161–93. doi: 10.3732/ajb.93.10.1512

[45] Hamukuaya H, O’Toole M, Woodhead P. Observations of severe hypoxia and offshore displacement of Cape Hake over the Namibian shelf in 1994. S Afr J Mar Sci. 1998;19(1):57–9.

[46] Bailey G, De B. Beyers C, Lipschitz S. Seasonal variation of oxygen deficiency in waters off southern South West Africa in 1975 and 1976 and its relation to the catchability and distribution of the Cape rock lobster Jasus lalandii. S Afr J Mar Sci. 1985;3(1):197–214.

[47] Currie B, Utne-Palm AC, Salvanes AGV. Winning ways with hydrogen sulphide on the Namibian shelf. Front Mar Sci. 2018;5. doi: 10.3389/fmars.2018.00341

[48] Brüchert V, Jørgensen B, Neumann K, Riechmann D, Schlösser M, Schulz H. Regulation of bacterial sulfate reduction and hydrogen sulfide fluxes in the central Namibian coastal upwelling zone. Geochim Cosmochim Acta. 2003;67(23):4505–18.

[49] McKay LJ, MacGregor BJ, Biddle JF, Albert DB, Mendlovitz HP, Hoer DR, et al. Spatial heterogeneity and underlying geochemistry of phylogenetically diverse orange and white *Beggiatoa* mats in Guaymas Basin hydrothermal sediments. Deep Sea Research Part I: Oceanographic Research Papers. 2012;67:21–31. doi: 10.1016/j.dsr.2012.04.011

[50] Grünke S, Felden J, Linchtschlag A, Girnth A-C, Beer DD, Enzhöfer FW, et al. Niche differentiation among mat-forming, sulfide-oxidizing bacteria at cold seeps of the Nile Deep Sea Fan (Eastern Mediterranean Sea). Geobiology. 2011;9(4):330–48. doi: 10.1111/j.1472-4669.2011.00281.x 21535364

[51] Bailey JV, Salman V, Rouse GW, Schulz-Vogt HN, Levin LA, Orphan VJ. Dimorphism in methane seep-dwelling ecotypes of the largest known bacteria. ISME J. 2011;5(12):1926–35. Epub 2011/06/24. doi: 10.1038/ismej.2011.66 ; PubMed Central PMCID: PMC3223306. 21697959 PMC3223306

[52] Barry JP, Greene GH, Orange DL, Baxter CH, Robison BH, Kochevar RE, et al. Biologic and geologic characteristics of cold seeps in Monterey Bay, California. Deep Sea Res Part I. 1996;43(11–12):1739–62.

[53] Gillan DC, Dubilier N. Novel epibiotic *Thiothrix* bacterium on a marine amphipod. Appl Environ Microbiol. 2004;70(6):3772–5. Epub 2004/06/09. doi: 10.1128/AEM.70.6.3772-3775.2004 ; PubMed Central PMCID: PMC427760. 15184190 PMC427760

[54] Mills HJ, Martinez RJ, Story S, Sobecky PA. Identification of members of the metabolically active microbial populations associated with *Beggiatoa* species mat communities from Gulf of Mexico cold-seep sediments. Appl Environ Microb. 2004;70(9):5447–58. Epub 2004/09/04. doi: 10.1128/AEM.70.9.5447-5458.2004 ; PubMed Central PMCID: PMC520919. 15345432 PMC520919

[55] Sharrar AM, Flood BE, Bailey JV, Jones DS, Biddanda BA, Ruberg SA, et al. Novel large sulfur bacteria in the metagenomes of groundwater-fed chemosynthetic microbial mats in the Lake Huron basin. Front Microbiol. 2017;8(791):791. Epub 2017/05/24. doi: 10.3389/fmicb.2017.00791 ; PubMed Central PMCID: PMC5421297. 28533768 PMC5421297

[56] Macalady JL, Lyon EH, Koffman B, Albertson LK, Meyer K, Galdenzi S, et al. Dominant microbial populations in limestone-corroding stream biofilms, Frasassi cave system, Italy. Appl Environ Microb. 2006;72(8):5596–609. Epub 2006/08/04. doi: 10.1128/AEM.00715-06 ; PubMed Central PMCID: PMC1538711. 16885314 PMC1538711

[57] Winkel M, Beer D, Lavik G, Peplies J, Mußmann M. Close association of active nitrifiers with *Beggiatoa* mats covering deep‐sea hydrothermal sediments. Environ Microbiol. 2014;16(6):1612–26. Epub 2013/11/30. doi: 10.1111/1462-2920.12316 . 24286252

[58] Prokopenko MG, Hirst MB, De Brabandere L, Lawrence D, Berelson W, Granger J, et al. Nitrogen losses in anoxic marine sediments driven by *Thioploca*-anammox bacterial consortia. Nature. 2013;500(7461):194–8. Epub 2013/08/09. doi: 10.1038/nature12365 . 23925243

[59] Schulz HN. The Genus Thiomargarita. The Prokaryotes 2006. p. 1156–63.

[60] Carpenter JH. The Chesapeake Bay Institute technique for the Winkler dissolved oxygen method. Limnol Oceanogr. 1965;10(1):141–3.

[61] Cline JD. Spectrophotometric determination of hydrogen sulfide in natural waters. Limnol Oceanogr. 1969;14(3):454–8.

[62] Gohl DM, Vangay P, Garbe J, MacLean A, Hauge A, Becker A, et al. Systematic improvement of amplicon marker gene methods for increased accuracy in microbiome studies. Nature Biotechnology. 2016;34(9):942–9. Epub 2016/07/28. doi: 10.1038/nbt.3601 . 27454739

[63] Walters W, Hyde ER, Berg-Lyons D, Ackermann G, Humphrey G, Parada A, et al. Improved bacterial 16s rrna gene (V4 and V4-5) and fungal internal transcribed spacer marker gene primers for microbial community surveys. mSystems. 2015;1(1). Epub 2016/11/09. doi: 10.1128/mSystems.00009-15 ; PubMed Central PMCID: PMC5069754. 27822518 PMC5069754

[64] Caporaso JG, Lauber CL, Walters WA, Berg-Lyons D, Lozupone CA, Turnbaugh PJ, et al. Global patterns of 16S rRNA diversity at a depth of millions of sequences per sample. Proc Natl Acad Sci. 2011;108:4516–22. doi: 10.1073/pnas.1000080107 20534432 PMC3063599

[65] Martin MW. Cutadapt removes adapter sequences from high-throughput sequencing reads. EMBnet Journal 2011;17:10–2.

[66] Callahan BJ, McMurdie PJ, Rosen MJ, Han AW, Johnson AJA, Holmes SP. DADA2: High-resolution sample inference from Illumina amplicon data. Nat Methods. 2016;13(7):581–3. Epub 2016/05/24. doi: 10.1038/nmeth.3869 ; PubMed Central PMCID: PMC4927377. 27214047 PMC4927377

[67] Quast C, Pruesse E, Yilmaz P, Gerken J, Schweer T, Yarza P, et al. The SILVA ribosomal RNA gene database project: improved data processing and web-based tools. Nucleic Acids Research. 2013;41(D1):D590–D6. Epub 2012/11/30. doi: 10.1093/nar/gks1219 ; PubMed Central PMCID: PMC3531112. 23193283 PMC3531112

[68] McMurdie PJ, Holmes S. Phyloseq: an R package for reproducible interactive analysis and graphics of microbiome census data. PLoS ONE. 2013;8(4):e61217. Epub 2013/05/01. doi: 10.1371/journal.pone.0061217 ; PubMed Central PMCID: PMC3632530. 23630581 PMC3632530

[69] Davis NM, Proctor DM, Holmes SP, Relman DA, Callahan BJ. Simple statistical identification and removal of contaminant sequences in marker-gene and metagenomics data. Microbiome. 2018;6(1):226. Epub 2018/12/19. doi: 10.1186/s40168-018-0605-2 ; PubMed Central PMCID: PMC6298009. 30558668 PMC6298009

[70] Love MI, Huber W, Anders S. Moderated estimation of fold change and dispersion for RNA-seq data with DESeq2. Genome Biology. 2014;15(12):550. Epub 2014/12/18. doi: 10.1186/s13059-014-0550-8 ; PubMed Central PMCID: PMC4302049. 25516281 PMC4302049

[71] Flohr A, Van der Plas A, Emeis K-C, Mohrholz V, Rixen T. Spatio-temporal patterns of C: N: P ratios in the northern Benguela upwelling system. Biogeosciences. 2014;11(3):885–97.

[72] Zoss R, Medina Ferrer F, Flood BE, Jones DS, Louw DC, Bailey J. Microbial communities associated with phosphogenic sediments and phosphoclast-associated DNA of the Benguela upwelling system. Geobiology. 2019;17(1):76–90. Epub 2018/10/29. doi: 10.1111/gbi.12318 . 30369004

[73] Salman V, Amann R, Shub DA, Schulz-Vogt HN. Multiple self-splicing introns in the 16S rRNA genes of giant sulfur bacteria. Proc Natl Acad Sci. 2012;109(11):4203–8. Epub 2012/03/01. doi: 10.1073/pnas.1120192109 ; PubMed Central PMCID: PMC3306719. 22371583 PMC3306719

[74] Puente-Sanchez F, Arce-Rodriguez A, Oggerin M, Garcia-Villadangos M, Moreno-Paz M, Blanco Y, et al. Viable cyanobacteria in the deep continental subsurface. Proc Natl Acad Sci U S A. 2018;115(42):10702–7. Epub 2018/10/03. doi: 10.1073/pnas.1808176115 ; PubMed Central PMCID: PMC6196553. 30275328 PMC6196553

[75] Nobu MK, Dodsworth JA, Murugapiran SK, Rinke C, Gies EA, Webster G, et al. Phylogeny and physiology of candidate phylum ‘Atribacteria’ (OP9/JS1) inferred from cultivation-independent genomics. ISME J. 2016;10(2):273–86. Epub 2015/06/20. doi: 10.1038/ismej.2015.97 ; PubMed Central PMCID: PMC4737943. 26090992 PMC4737943

[76] Lee YM, Hwang K, Lee JI, Kim M, Hwang CY, Noh H-J, et al. Genomic insight Into the predominance of candidate phylum Atribacteria JS1 lineage in marine sediments. Front Microbiol. 2018;9:2909. Epub 2018/12/18. doi: 10.3389/fmicb.2018.02909 ; PubMed Central PMCID: PMC6281690. 30555444 PMC6281690

[77] Jacobson ME, Mackin JE, Capone DG. Ammonium production in sediments inhibited with molybdate: Implications for the sources of ammonium in anoxic marine sediments. Appl Environ Microb. 1987;53(10):2435–9. Epub 1987/10/01. doi: 10.1128/aem.53.10.2435-2439.1987 ; PubMed Central PMCID: PMC204125. 16347462 PMC204125

[78] Kraft B, Tegetmeyer HE, Sharma R, Klotz MG, Ferdelman TG, Hettich RL, et al. The environmental controls that govern the end product of bacterial nitrate respiration. Science. 2014;345(6197):676–9. Epub 2014/08/12. doi: 10.1126/science.1254070 . 25104387

[79] Devol AH. Denitrification, anammox, and N(2) production in marine sediments. Ann Rev Mar Sci. 2015;7:403–23. Epub 2015/01/07. doi: 10.1146/annurev-marine-010213-135040 . 25560607

[80] Rios-Del Toro EE, Cervantes FJ. Coupling between anammox and autotrophic denitrification for simultaneous removal of ammonium and sulfide by enriched marine sediments. Biodegradation. 2016;27(2–3):107–18. Epub 2016/03/21. doi: 10.1007/s10532-016-9759-4 . 26994921

[81] Pajares S, Ramos R. Processes and microorganisms involved in the marine nitrogen cycle: Knowledge and gaps. Front Mar Sci. 2019;6. doi: 10.3389/fmars.2019.00739

[82] Fincker M, Huber JA, Orphan VJ, Rappe MS, Teske A, Spormann AM. Metabolic strategies of marine subseafloor Chloroflexi inferred from genome reconstructions. Environ Microbiol. 2020;22(8):3188–204. Epub 2020/05/07. doi: 10.1111/1462-2920.15061 . 32372496

[83] Rinke C, Rubino F, Messer LF, Youssef N, Parks DH, Chuvochina M, et al. A phylogenomic and ecological analysis of the globally abundant Marine Group II archaea (Ca. Poseidoniales ord. nov.). ISME J. 2019;13(3):663–75. Epub 2018/10/17. doi: 10.1038/s41396-018-0282-y ; PubMed Central PMCID: PMC6461757. 30323263 PMC6461757

[84] Lloyd KG, Schreiber L, Petersen DG, Kjeldsen KU, Lever MA, Steen AD, et al. Predominant archaea in marine sediments degrade detrital proteins. Nature. 2013;496(7444):215–8. Epub 2013/03/29. doi: 10.1038/nature12033 . 23535597

[85] Cardich J, Gutiérrez D, Romero D, Pérez A, Quipúzcoa L, Marquina R, et al. Calcareous benthic foraminifera from the upper central Peruvian margin: control of the assemblage by pore water redox and sedimentary organic matter. Marine Ecology Progress Series. 2015;535:63–87.

[86] Gutiérrez D, Enriquez E, Purca S, Quipúzcoa L, Marquina R, Flores G, et al. Oxygenation episodes on the continental shelf of central Peru: Remote forcing and benthic ecosystem response. Prog Oceanogr. 2008;79(2–4):177–89.

[87] Brüchert V, Currie B, Peard KR. Hydrogen sulphide and methane emissions on the central Namibian shelf. Prog Oceanogr. 2009;83(1–4):169–79. doi: 10.1016/j.pocean.2009.07.017

[88] Dale AW, Brüchert V, Alperin M, Regnier P. An integrated sulfur isotope model for Namibian shelf sediments. Geochim Cosmochim Acta. 2009;73(7):1924–44.

[89] Schulz HN, Schulz HD. Large sulfur bacteria and the formation of phosphorite. Nature. 2005;307(5708):416–8. Epub 2005/01/22. doi: 10.1126/science.1103096 . 15662012

[90] Emeis K-C, Brüchert V, Currie B, Endler R, Ferdelman T, Kiessling A, et al. Shallow gas in shelf sediments of the Namibian coastal upwelling ecosystem. Cont Shelf Res. 2004;24:627–4.

[91] Fossing H, Ferdelman TG, Berg P. Sulfate reduction and methane oxidation in continental margin sediments influenced by irrigation (South-East Atlantic off Namibia). Geochim Cosmochim Acta. 2000;64(5):897–910.

[92] Neumann A, Lahajnar N, Emeis K-C. Benthic remineralisation rates in shelf and slope sediments of the northern Benguela upwelling margin. Cont Shelf Res. 2016;113:47–61. doi: 10.1016/j.csr.2015.12.009

[93] Evans TW, Wörmer L, Lever MA, Lipp JS, Lagostina L, Lin Y-S, et al. Size and composition of subseafloor microbial community in the Benguela upwelling area examined from intact membrane lipid and DNA analysis. Organic Geochemistry. 2017;111:86–100. doi: 10.1016/j.orggeochem.2017.06.008

[94] Goldhammer T, Brunner B, Bernasconi SM, Ferdelman TG, Zabel M. Phosphate oxygen isotopes: Insights into sedimentary phosphorus cycling from the Benguela upwelling system. Geochim Cosmochim Acta. 2011;75(13):3741–56. doi: 10.1016/j.gca.2011.04.006

[95] Qin W, Meinhardt KA, Moffett JW, Devol AH, Virginia Armbrust E, Ingalls AE, et al. Influence of oxygen availability on the activities of ammonia-oxidizing archaea. Environ Microbiol Rep. 2017;9(3):250–6. Epub 2017/02/18. doi: 10.1111/1758-2229.12525 . 28211189

[96] Stewart FJ, Ulloa O, DeLong EF. Microbial metatranscriptomics in a permanent marine oxygen minimum zone. Environ Microbiol. 2012;14(1):23–40. Epub 2011/01/08. doi: 10.1111/j.1462-2920.2010.02400.x . 21210935

[97] Füssel J, Lücker S, Yilmaz P, Nowka B, van Kessel MA, Bourceau P, et al. Adaptability as the key to success for the ubiquitous marine nitrite oxidizer *Nitrococcus*. Sci Adv. 2017;3(11):e1700807. doi: 10.1126/sciadv.1700807 29109973 PMC5665590

[98] Teske A, Jørgensen BB, Gallardo VA. Filamentous bacteria inhabiting the sheaths of marine *Thioploca* spp.on the Chilean continental shelf. FEMS Microbiol Ecol. 2010;68:164–72. 10.1111/j.1574-6941.2009.00659.x 19573198

[99] Watanabe M, Kojima H, Umezawa K, Fukui M. Genomic characteristics of *Desulfonema ishimotonii* tokyo 01(T) implying horizontal gene transfer among phylogenetically dispersed filamentous gliding bacteria. Front Microbiol. 2019;10:227. Epub 2019/03/07. doi: 10.3389/fmicb.2019.00227 ; PubMed Central PMCID: PMC6390638. 30837965 PMC6390638

[100] Watsuji T-O, Nishizawa M, Morono Y, Hirayama H, Kawagucci S, Takahata N, et al. Cell-specific thioautotrophic productivity of Epsilon-proteobacterial epibionts associated with *Shinkaia crosnieri*. PLoS ONE. 2012;7(10):e46282. Epub 2012/10/12. doi: 10.1371/journal.pone.0046282 ; PubMed Central PMCID: PMC3462759. 23056274 PMC3462759

[101] Stokke R, Dahle H, Roalkvam I, Wissuwa J, Daae FL, Tooming‐Klunderud A, et al. Functional interactions among filamentous Epsilonproteobacteria and Bacteroidetes in a deep‐sea hydrothermal vent biofilm. Environ Microbiol. 2015;17(10):4063–77. doi: 10.1111/1462-2920.12970 26147346

[102] Buck KR, Barry JP, Hallam SJ. *Thioploca* spp. sheaths as niches for bacterial and protistan assemblages. Marine Ecology. 2014;35(3):395–400. doi: 10.1111/maec.12076

[103] Lage OM, Bondoso J. Planctomycetes and macroalgae, a striking association. Front Microbiol. 2014;5:267. Epub 2014/06/12. doi: 10.3389/fmicb.2014.00267 ; PubMed Central PMCID: PMC4042473. 24917860 PMC4042473

[104] Woebken D, Teeling H, Wecker P, Dumitriu A, Kostadinov I, Delong EF, et al. Fosmids of novel marine Planctomycetes from the Namibian and Oregon coast upwelling systems and their cross-comparison with planctomycete genomes. ISME J. 2007;1(5):419–35. Epub 2007/11/29. doi: 10.1038/ismej.2007.63 . 18043661

[105] Stevens E, Bailey J, Flood B, Jones D, Gilhooly W, Joye S, et al. Barite encrustation of benthic sulfur‐oxidizing bacteria at a marine cold seep. Geobiology. 2015;13(6):588–603. doi: 10.1111/gbi.12154 26462132

[106] van Vliet DM, Palakawong Na Ayudthaya S, Diop S, Villanueva L, Stams AJM, Sanchez-Andrea I. Anaerobic degradation of sulfated polysaccharides by two novel Kiritimatiellales strains isolated from Black Sea sediment. Front Microbiol. 2019;10:253. Epub 2019/03/06. doi: 10.3389/fmicb.2019.00253 ; PubMed Central PMCID: PMC6388578. 30833937 PMC6388578

[107] Youssef NH, Farag IF, Rinke C, Hallam SJ, Woyke T, Elshahed MS. In silico analysis of the metabolic potential and niche specialization of candidate phylum “Latescibacteria" (WS3). PLoS ONE. 2015;10(6):e0127499. Epub 2015/06/04. doi: 10.1371/journal.pone.0127499 ; PubMed Central PMCID: PMC4454575. 26039074 PMC4454575

[108] Marshall IPG, Starnawski P, Cupit C, Fernandez Caceres E, Ettema TJG, Schramm A, et al. The novel bacterial phylum Calditrichaeota is diverse, widespread and abundant in marine sediments and has the capacity to degrade detrital proteins. Environ Microbiol Rep. 2017;9(4):397–403. Epub 2017/05/11. doi: 10.1111/1758-2229.12544 . 28488795

[109] Amiri Moghaddam J, Crusemann M, Alanjary M, Harms H, Davila-Cespedes A, Blom J, et al. Analysis of the genome and metabolome of marine Myxobacteria reveals high potential for biosynthesis of novel specialized metabolites. Sci Rep. 2018;8(1):16600. Epub 2018/11/11. doi: 10.1038/s41598-018-34954-y ; PubMed Central PMCID: PMC6226438. 30413766 PMC6226438

[110] Kielak AM, Barreto CC, Kowalchuk GA, van Veen JA, Kuramae EE. The ecology of Acidobacteria: Moving beyond genes and genomes. Front Microbiol. 2016;7:744. Epub 2016/06/16. doi: 10.3389/fmicb.2016.00744 ; PubMed Central PMCID: PMC4885859. 27303369 PMC4885859

[111] Dubinina G, Grabovich M, Leshcheva N, Rainey FA, Gavrish E. *Spirochaeta perfilievii* sp. nov., an oxygen-tolerant, sulfide-oxidizing, sulfur- and thiosulfate-reducing spirochaete isolated from a saline spring. Int J Syst Evol Microbiol. 2011;61(Pt 1):110–7. Epub 2010/02/23. doi: 10.1099/ijs.0.018333-0 . 20173011

[112] Temuujin U, Chi WJ, Park JS, Chang YK, Song JY, Hong SK. Identification and characterization of a novel beta-galactosidase from *Victivallis vadensis* ATCC BAA-548, an anaerobic fecal bacterium. J Microbiol. 2012;50(6):1034–40. Epub 2013/01/01. doi: 10.1007/s12275-012-2478-6 . 23274992

[113] Doud DFR, Bowers RM, Schulz F, De Raad M, Deng K, Tarver A, et al. Function-driven single-cell genomics uncovers cellulose-degrading bacteria from the rare biosphere. ISME J. 2020;14(3):659–75. Epub 2019/11/23. doi: 10.1038/s41396-019-0557-y ; PubMed Central PMCID: PMC7031533. 31754206 PMC7031533

[114] Saad S, Bhatnagar S, Tegetmeyer HE, Geelhoed JS, Strous M, Ruff SE. Transient exposure to oxygen or nitrate reveals ecophysiology of fermentative and sulfate-reducing benthic microbial populations. Environ Microbiol. 2017;19(12):4866–81. Epub 2017/08/25. doi: 10.1111/1462-2920.13895 ; PubMed Central PMCID: PMC5763382. 28836729 PMC5763382

[115] Coskun ÖK, Pichler M, Vargas S, Gilder S, Orsi WD. Linking uncultivated microbial populations and benthic carbon turnover by using quantitative stable isotope probing. Appl Environ Microb. 2018;84(18). doi: 10.1128/AEM.01083-18 29980553 PMC6122004

[116] Utami YD, Kuwahara H, Murakami T, Morikawa T, Sugaya K, Kihara K, et al. Phylogenetic diversity and single-cell genome analysis of “Melainabacteria", a non-photosynthetic cyanobacterial group, in the termite gut. Microbes Environ. 2018;33(1):50–7. Epub 2018/02/09. doi: 10.1264/jsme2.ME17137 ; PubMed Central PMCID: PMC5877343. 29415909 PMC5877343

[117] Louca S, Parfrey LW, Doebeli M. Decoupling function and taxonomy in the global ocean microbiome. Science. 2016;353(6305):1272–7. Epub 2016/09/17. doi: 10.1126/science.aaf4507 . 27634532

[118] Sekiguchi Y, Ohashi A, Parks DH, Yamauchi T, Tyson GW, Hugenholtz P. First genomic insights into members of a candidate bacterial phylum responsible for wastewater bulking. PeerJ. 2015;3:e740. Epub 2015/02/05. doi: 10.7717/peerj.740 ; PubMed Central PMCID: PMC4312070. 25650158 PMC4312070

[119] Castelle CJ, Hug LA, Wrighton KC, Thomas BC, Williams KH, Wu D, et al. Extraordinary phylogenetic diversity and metabolic versatility in aquifer sediment. Nat Commun. 2013;4:2120. Epub 2013/08/28. doi: 10.1038/ncomms3120 ; PubMed Central PMCID: PMC3903129. 23979677 PMC3903129

[120] Isolation Dyar M. and cytological study of a free-living spirochete. J Bacteriol. 1947;54(4):483–93. doi: 10.1128/jb.54.4.483-493.1947 16561383 PMC526578

[121] Blakemore R, Canale-Parola E. Morphological and ecological characteristics of *Spirochaeta plicatilis*. Archiv für Mikrobiologie. 1973;89(4):273–89. https://doi-org.ezp2.lib.umn.edu/10.1007/BF00408895. 4571290 10.1007/BF00408895

[122] Grabovich MY, Gureeva MV, Dubinina GA. The role of the "*Thiodendron*" consortium in postulating the karyomastigont chimaera of the endosymbiosis theory by Lynn Margulis. Biosystems. 2021;200:104322. Epub 2020/12/15. doi: 10.1016/j.biosystems.2020.104322 . 33309968

[123] Romine MF, Rodionov DA, Maezato Y, Osterman AL, Nelson WC. Underlying mechanisms for syntrophic metabolism of essential enzyme cofactors in microbial communities. ISME J. 2017;11(6):1434–46. Epub 2017/02/12. doi: 10.1038/ismej.2017.2 ; PubMed Central PMCID: PMC5437353. 28186498 PMC5437353

[124] Tian R, Ning D, He Z, Zhang P, Spencer SJ, Gao S, et al. Small and mighty: adaptation of superphylum Patescibacteria to groundwater environment drives their genome simplicity. Microbiome. 2020;8(1):51. Epub 2020/04/08. doi: 10.1186/s40168-020-00825-w ; PubMed Central PMCID: PMC7137472. 32252814 PMC7137472

[125] Herrmann M, Wegner CE, Taubert M, Geesink P, Lehmann K, Yan L, et al. Predominance of *Cand*. Patescibacteria in groundwater is caused by their preferential mobilization from soils and flourishing under oligotrophic conditions. Front Microbiol. 2019;10:1407. Epub 2019/07/10. doi: 10.3389/fmicb.2019.01407 ; PubMed Central PMCID: PMC6596338. 31281301 PMC6596338

[126] Liu X, Li M, Castelle CJ, Probst AJ, Zhou Z, Pan J, et al. Insights into the ecology, evolution, and metabolism of the widespread Woesearchaeotal lineages. Microbiome. 2018;6(1):102. Epub 2018/06/10. doi: 10.1186/s40168-018-0488-2 ; PubMed Central PMCID: PMC5994134. 29884244 PMC5994134

[127] Biebl H, Pfennig N. Growth yields of green sulfur bacteria in mixed cultures with sulfur and sulfate reducing bacteria. Arch Micro. 1978;117(1):9–16.

[128] Kaden J, Galushko AS, Schink B. Cysteine-mediated electron transfer in syntrophic acetate oxidation by cocultures of *Geobacter sulfurreducens* and *Wolinella succinogenes*. Arch Micro. 2002;178(1):53–8. Epub 2002/06/19. doi: 10.1007/s00203-002-0425-3 . 12070769

[129] van den Ende FP, Meier J, van Gemerden H. Syntrophic growth of sulfate-reducing bacteria and colorless sulfur bacteria during oxygen limitation. FEMS Microbiol Ecol. 1997;23:65–80.

[130] Schreiber L, Holler T, Knittel K, Meyerdierks A, Amann R. Identification of the dominant sulfate-reducing bacterial partner of anaerobic methanotrophs of the ANME-2 clade. Environ Microbiol. 2010;12(8):2327–40. Epub 2010/08/01. doi: 10.1111/j.1462-2920.2010.02275.x . 21966923

[131] Chen SC, Musat N, Lechtenfeld OJ, Paschke H, Schmidt M, Said N, et al. Anaerobic oxidation of ethane by archaea from a marine hydrocarbon seep. Nature. 2019;568(7750):108–11. Epub 2019/03/29. doi: 10.1038/s41586-019-1063-0 . 30918404

[132] Kendall MM, Liu Y, Boone DR. Butyrate- and propionate-degrading syntrophs from permanently cold marine sediments in Skan Bay, Alaska, and description of *Algorimarina butyrica* gen. nov., sp. nov. FEMS Microbiol Lett. 2006;262(1):107–14. Epub 2006/08/16. doi: 10.1111/j.1574-6968.2006.00380.x . 16907746

[133] Salonen IS, Chronopoulou P, Bird C, Reichart G, Koho KA. Enrichment of intracellular sulphur cycle -associated bacteria in intertidal benthic foraminifera revealed by 16S and aprA gene analysis. Sci Rep. 2019;9(1):11692. Epub 2019/08/14. doi: 10.1038/s41598-019-48166-5 ; PubMed Central PMCID: PMC6690927. 31406214 PMC6690927

[134] Beinart RA, Beaudoin DJ, Bernhard JM, Edgcomb VP. Insights into the metabolic functioning of a multipartner ciliate symbiosis from oxygen-depleted sediments. Mol Ecol. 2018;27(8):1794–807. Epub 2017/12/23. doi: 10.1111/mec.14465 . 29271011

[135] Li M, Baker BJ, Anantharaman K, Jain S, Breier JA, Dick GJ. Genomic and transcriptomic evidence for scavenging of diverse organic compounds by widespread deep-sea archaea. Nat Commun. 2015;6:8933. Epub 2015/11/18. doi: 10.1038/ncomms9933 ; PubMed Central PMCID: PMC4660358. 26573375 PMC4660358

[136] Zinke LA, Evans PN, Schroeder AL, Parks DH, Varner RK, Rich VI, et al. Evidence for non-methanogenic metabolisms in globally distributed archaeal clades basal to the Methanomassiliicoccales. Environ Microbiol. 2020;23(1):340–57. Epub 2020/11/14. doi: 10.1111/1462-2920.15316 . 33185945

[137] Kolinko S, Richter M, Glöckner F-O, Brachmann A, Schüler D. Single-cell genomics of uncultivated deep-branching magnetotactic bacteria reveals a conserved set of magnetosome genes. Environ Microbiol. 2016;18(1):21–37. Epub 2015/06/11. doi: 10.1111/1462-2920.12907 . 26060021

[138] Sheik CS, Jain S, Dick GJ. Metabolic flexibility of enigmatic SAR 324 revealed through metagenomics and metatranscriptomics. Environ Microbiol. 2014;16(1):304–17. doi: 10.1111/1462-2920.12165 23809230

[139] Dyksma S, Bischof K, Fuchs BM, Hoffmann K, Meier D, Meyerdierks A, et al. Ubiquitous Gammaproteobacteria dominate dark carbon fixation in coastal sediments. ISME J. 2016;10(8):1939–53. Epub 2016/02/13. doi: 10.1038/ismej.2015.257 ; PubMed Central PMCID: PMC4872838. 26872043 PMC4872838

[140] Almenglo F, Bezerra T, Lafuente J, Gabriel D, Ramirez M, Cantero D. Effect of gas-liquid flow pattern and microbial diversity analysis of a pilot-scale biotrickling filter for anoxic biogas desulfurization. Chemosphere. 2016;157:215–23. Epub 2016/05/28. doi: 10.1016/j.chemosphere.2016.05.016 . 27231880

[141] Xu AOX-W. 11 The Family Hyphomicrobiaceae. 2014.

[142] Lenk S, Moraru C, Hahnke S, Arnds J, Richter M, Kube M, et al. Roseobacter clade bacteria are abundant in coastal sediments and encode a novel combination of sulfur oxidation genes. ISME J. 2012;6(12):2178–87. Epub 2012/06/29. doi: 10.1038/ismej.2012.66 ; PubMed Central PMCID: PMC3504970. 22739490 PMC3504970

[143] Moran MA, Durham BP. Sulfur metabolites in the pelagic ocean. Nat Rev Microbiol. 2019;17(11):665–78. Epub 2019/09/06. doi: 10.1038/s41579-019-0250-1 . 31485034

[144] Han Y, Perner M. The globally widespread genus *Sulfurimonas*: versatile energy metabolisms and adaptations to redox clines. Front Microbiol. 2015;6:989. Epub 2015/10/07. doi: 10.3389/fmicb.2015.00989 ; PubMed Central PMCID: PMC4584964. 26441918 PMC4584964

[145] Patwardhan S, Foustoukos DI, Giovannelli D, Yucel M, Vetriani C. Ecological succession of sulfur-oxidizing Epsilon- and Gammaproteobacteria during colonization of a shallow-water gas vent. Front Microbiol. 2018;9:2970. Epub 2018/12/24. doi: 10.3389/fmicb.2018.02970 ; PubMed Central PMCID: PMC6291522. 30574130 PMC6291522

[146] O’Brien CE, Giovannelli D, Govenar B, Luther GW, Lutz RA, Shank TM, et al. Microbial biofilms associated with fluid chemistry and megafaunal colonization at post-eruptive deep-sea hydrothermal vents. Deep-Sea Res Pt II. 2015;121:31–40. doi: 10.1016/j.dsr2.2015.07.020

[147] Meier DV, Pjevac P, Bach W, Hourdez S, Girguis PR, Vidoudez C, et al. Niche partitioning of diverse sulfur-oxidizing bacteria at hydrothermal vents. ISME J. 2017;11(7):1545–58. Epub 2017/04/05. doi: 10.1038/ismej.2017.37 ; PubMed Central PMCID: PMC5520155. 28375213 PMC5520155

[148] Macalady JL, Dattagupta S, Schaperdoth I, Jones DS, Druschel GK, Eastman D. Niche differentiation among sulfur-oxidizing bacterial populations in cave waters. ISME J. 2008;2(6):590–601. Epub 2008/03/22. doi: 10.1038/ismej.2008.25 . 18356823

[149] Sievert SM, Wieringa EB, Wirsen CO, Taylor CD. Growth and mechanism of filamentous-sulfur formation by *Candidatus* Arcobacter sulfidicus in opposing oxygen-sulfide gradients. Environ Microbiol. 2007;9(1):271–6. Epub 2007/01/18. doi: 10.1111/j.1462-2920.2006.01156.x . 17227432

[150] Hamilton TL, Jones DS, Schaperdoth I, Macalady JL. Metagenomic insights into S(0) precipitation in a terrestrial subsurface lithoautotrophic ecosystem. Front Microbiol. 2014;5:756. Epub 2015/01/27. doi: 10.3389/fmicb.2014.00756 ; PubMed Central PMCID: PMC4288042. 25620962 PMC4288042

[151] Pjevac P, Kamyshny A Jr., Dyksma S, Mussmann M. Microbial consumption of zero-valence sulfur in marine benthic habitats. Environ Microbiol. 2014;16(11):3416–30. Epub 2014/01/29. doi: 10.1111/1462-2920.12410 . 24467476

[152] Gotz F, Pjevac P, Markert S, McNichol J, Becher D, Schweder T, et al. Transcriptomic and proteomic insight into the mechanism of cyclooctasulfur- versus thiosulfate-oxidation by the chemolithoautotroph *Sulfurimonas denitrificans*. Environ Microbiol. 2019;21(1):244–58. Epub 2018/10/27. doi: 10.1111/1462-2920.14452 . 30362214

[153] Wang S, Jiang L, Hu Q, Liu X, Yang S, Shao Z. Elemental sulfur reduction by a deep-sea hydrothermal vent Campylobacterium *Sulfurimonas* sp. NW10. Environ Microbiol. 2020. Epub 2020/09/26. doi: 10.1111/1462-2920.15247 32974951

[154] Makita H, Nakagawa S, Miyazaki M, Nakamura K-i, Inagaki F, Takai K. *Thiofractor thiocaminus* gen. nov., sp. nov., a novel hydrogen-oxidizing, sulfur-reducing epsilonproteobacterium isolated from a deep-sea hydrothermal vent chimney in the Nikko Seamount field of the northern Mariana Arc. Arch Micro. 2012;194(9):785–94. Epub 2012/04/25. doi: 10.1007/s00203-012-0814-1 . 22526267

[155] Mino S, Kudo H, Arai T, Sawabe T, Takai K, Nakagawa S. *Sulfurovum aggregans* sp. nov., a hydrogen-oxidizing, thiosulfate-reducing chemolithoautotroph within the Epsilonproteobacteria isolated from a deep-sea hydrothermal vent chimney, and an emended description of the genus Sulfurovum. Int J Syst Evol Microbiol. 2014;64(Pt 9):3195–201. Epub 2014/06/27. doi: 10.1099/ijs.0.065094-0 . 24966202

[156] Jørgensen BB, Revsbech NP. Colorless sulfur bacteria, *Beggiatoa* spp. and *Thiovulum* spp., in O2 and H2S microgradients. Appl Environ Microb. 1983;45(4):1261–70. 10.1128/aem.45.4.1261-1270.1983 PMC242448 16346268

[157] Mishanina TV, Libiad M, Banerjee R. Biogenesis of reactive sulfur species for signaling by hydrogen sulfide oxidation pathways. Nat Chem Biol. 2015;11(7):457–64. Epub 2015/06/18. doi: 10.1038/nchembio.1834 ; PubMed Central PMCID: PMC4818113. 26083070 PMC4818113

[158] Tanabe TS, Leimkuhler S, Dahl C. The functional diversity of the prokaryotic sulfur carrier protein TusA. Adv Microb Physiol. 2019;75:233–77. Epub 2019/10/28. doi: 10.1016/bs.ampbs.2019.07.004 . 31655739

[159] Böttcher ME, Thamdrup B, Gehre M, Theune A. 34S/32S and18O/16O fractionation during sulfur disproportionation by *Desulfobulbus propionicus*. Geomicrobiol J. 2005;22(5):219–26. doi: 10.1080/01490450590947751

[160] Slobodkin AI, Slobodkina GB. Diversity of sulfur-disproportionating microorganisms. Microbiology. 2019;88(5):509–22. doi: 10.1134/s0026261719050138

[161] Zhao R, Hannisdal B, Mogollon JM, Jorgensen SL. Nitrifier abundance and diversity peak at deep redox transition zones. Sci Rep. 2019;9(1):8633. Epub 2019/06/16. doi: 10.1038/s41598-019-44585-6 ; PubMed Central PMCID: PMC6572806. 31201353 PMC6572806

[162] Koch H, van Kessel M, Lucker S. Complete nitrification: insights into the ecophysiology of comammox *Nitrospira*. Appl Microbiol Biotechnol. 2019;103(1):177–89. Epub 2018/11/12. doi: 10.1007/s00253-018-9486-3 ; PubMed Central PMCID: PMC6311188. 30415428 PMC6311188

[163] Daims H, Lebedeva EV, Pjevac P, Han P, Herbold C, Albertsen M, et al. Complete nitrification by *Nitrospira* bacteria. Nature. 2015;528(7583):504–9. Epub 2015/11/27. doi: 10.1038/nature16461 ; PubMed Central PMCID: PMC5152751. 26610024 PMC5152751

[164] Park S-J, Andrei A-Ş, Bulzu P-A, Kavagutti VS, Ghai R, Mosier AC. Expanded diversity and metabolic versatility of marine nitrite-oxidizing bacteria revealed by cultivation-and genomics-based approaches. Appl Environ Microb. 2020;86(22). 10.1128/AEM.01667-20 PMC7642081 32917751

[165] Sun X, Kop LFM, Lau MCY, Frank J, Jayakumar A, Lucker S, et al. Uncultured *Nitrospina*-like species are major nitrite oxidizing bacteria in oxygen minimum zones. ISME J. 2019;13(10):2391–402. Epub 2019/05/24. doi: 10.1038/s41396-019-0443-7 ; PubMed Central PMCID: PMC6776041. 31118472 PMC6776041

[166] Kalnins M, Berzins A, Gudra D, Megnis K, Fridmanis D, Danilko P, et al. Selective enrichment of heterotrophic nitrifiers Alcaligenaceae and *Alcanivorax* spp. from industrial wastewaters. AIMS Microbiol. 2020;6(1):32–42. Epub 2020/04/01. doi: 10.3934/microbiol.2020002 ; PubMed Central PMCID: PMC7099200. 32226913 PMC7099200

[167] Liu X, Shu Z, Sun D, Dang Y, Holmes DE. Heterotrophic nitrifiers dominate reactors treating incineration leachate with high free ammonia concentrations. ACS Sustain Chem Eng. 2018;6(11):15040–9. doi: 10.1021/acssuschemeng.8b03512

[168] Mori JF, Chen LX, Jessen GL, Rudderham SB, McBeth JM, Lindsay MBJ, et al. Putative mixotrophic nitrifying-denitrifying Gammaproteobacteria implicated in nitrogen cycling within the ammonia/oxygen transition zone of an oil sands pit lake. Front Microbiol. 2019;10:2435. Epub 2019/11/12. doi: 10.3389/fmicb.2019.02435 ; PubMed Central PMCID: PMC6824324. 31708903 PMC6824324

[169] Wehrfritz J-M, Carter JP, Spiro S, Richardson DJ. Hydroxylamine oxidation in heterotrophic nitrate-reducing soil bacteria and purification of a hydroxylamine-cytochromec oxidoreductase from a *Pseudomonas* species. Arch Micro. 1996;166(6):421–4. doi: 10.1007/BF01682991 9082922

[170] Wehrfritz J-M, Reilly A, Spiro S, Richardson DJ. Purification of hydroxylamine oxidase from *Thiosphaera pantotropha*: Identification of electron acceptors that couple heterotrophic nitrification to aerobic denitrification. FEBS letters. 1993;335(2):246–50. doi: 10.1016/0014-5793(93)80739-h 8253206

[171] Moir JW, Wehrfritz J-M, Sppiro S, Richardson DJ. The biochemical characterization of a novel non-haem-iron hydroxylamine oxidase from *Paracoccus denitrificans* GB17. Biochemical Journal. 1996;319(3):823–7. Epub 1996/11/01. doi: 10.1042/bj3190823 ; PubMed Central PMCID: PMC1217862. 8920986 PMC1217862

[172] Moir JW, Crossman LC, Spiro S, Richardson DJ. The purification of ammonia monooxygenase from *Paracoccus denitrficans*. FEBS Letters. 1996;387(1):71–4. doi: 10.1016/0014-5793(96)00463-2 8654570

[173] Stein LY. Heterotrophic nitrification and nitrifier denitrification. Nitrification. 2011:95–114.

[174] Robertson LA, Kuenen JG. Combined heterotrophic nitrification and aerobic denitrification in Thiosphaera pantotropha and other bacteria. Antonie Leeuwenhoek. 1990;57(3):139–52. Epub 1990/04/01. doi: 10.1007/BF00403948 2181927

[175] Schmidt I, Bock E. Anaerobic ammonia oxidation with nitrogen dioxide by *Nitrosomonas eutropha*. Arch Micro. 1997;167(2–3):106–11. Epub 1997/02/01. 9133316

[176] Kozlowski JA, Kits KD, Stein LY. Comparison of nitrogen oxide metabolism among diverse ammonia-oxidizing bacteria. Front Microbiol. 2016;7:1090. Epub 2016/07/28. doi: 10.3389/fmicb.2016.01090 ; PubMed Central PMCID: PMC4940428. 27462312 PMC4940428

[177] Kozlowski JA, Stieglmeier M, Schleper C, Klotz MG, Stein LY. Pathways and key intermediates required for obligate aerobic ammonia-dependent chemolithotrophy in bacteria and Thaumarchaeota. ISME J. 2016;10(8):1836–45. Epub 2016/02/18. doi: 10.1038/ismej.2016.2 ; PubMed Central PMCID: PMC5029154. 26882267 PMC5029154

[178] Liu S, Han P, Hink L, Prosser JI, Wagner M, Bruggemann N. Abiotic conversion of extracellular NH2OH contributes to N2O emission during ammonia oxidation. Environ Sci Technol. 2017;51(22):13122–32. Epub 2017/10/19. doi: 10.1021/acs.est.7b02360 . 29039187

[179] Goreau TJ, Kaplan WA, Wofsy SC, McElroy MB, Valois FW, Watson SW. Production of NO2-and N2O by nitrifying bacteria at reduced concentrations of oxygen. Appl Environ Microb. 1980;40(3):526–32. 10.1128/aem.40.3.526-532.1980 PMC291617 16345632

[180] Bock E, Schmidt I, Stüven R, Zart D. Nitrogen loss caused by denitrifying *Nitrosomonas* cells using ammonium or hydrogen as electron donors and nitrite as electron acceptor. Arch Micro. 1995;163(1):16–20.

[181] Schrum HN, Spivack AJ, Kastner M, D’Hondt S. Sulfate-reducing ammonium oxidation: A thermodynamically feasible metabolic pathway in subseafloor sediment. Geology. 2009;37(10):939–42. doi: 10.1130/g30238a.1

[182] Yang Z, Zhou S, Sun Y. Start-up of simultaneous removal of ammonium and sulfate from an anaerobic ammonium oxidation (anammox) process in an anaerobic up-flow bioreactor. J Hazard Mater. 2009;169(1–3):113–8. Epub 2009/04/29. doi: 10.1016/j.jhazmat.2009.03.067 . 19398270

[183] Liu LY, Xie GJ, Xing DF, Liu BF, Ding J, Cao GL, et al. Sulfate dependent ammonium oxidation: A microbial process linked nitrogen with sulfur cycle and potential application. Environ Res. 2021;192:110282. Epub 2020/10/11. doi: 10.1016/j.envres.2020.110282 . 33038361

[184] Rios-Del Toro EE, Valenzuela EI, Lopez-Lozano NE, Cortes-Martinez MG, Sanchez-Rodriguez MA, Calvario-Martinez O, et al. Anaerobic ammonium oxidation linked to sulfate and ferric iron reduction fuels nitrogen loss in marine sediments. Biodegradation. 2018;29(5):429–42. Epub 2018/06/28. doi: 10.1007/s10532-018-9839-8 . 29948518

[185] Fdz-Polanco F, Fdz-Polanco M, Fernandez N, Urueña M, Garcia P, Villaverde S. Combining the biological nitrogen and sulfur cycles in anaerobic conditions. Water Sci Technol. 2001;44(8):77–84. Epub 2001/12/04. . 11730140

[186] Zhang D, Cui L, Wang H, Liang J. Study of sulfate-reducing ammonium oxidation process and its microbial community composition. Water Sci Technol. 2019;79(1):137–44. Epub 2019/03/01. doi: 10.2166/wst.2019.027 ; PubMed Central PMCID: PMCwst_2019_027. 30816870

[187] Rikmann E, Zekker I, Tomingas M, Tenno T, Menert A, Loorits L, et al. Sulfate-reducing anaerobic ammonium oxidation as a potential treatment method for high nitrogen-content wastewater. Biodegradation. 2012;23(4):509–24. Epub 2011/12/30. doi: 10.1007/s10532-011-9529-2 . 22205544

[188] Rikmann E, Zekker I, Tomingas M, Tenno T, Loorits L, Vabamäe P, et al. Sulfate-reducing anammox for sulfate and nitrogen containing wastewaters. Desalination Water Treat. 2014;57(7):3132–41. doi: 10.1080/19443994.2014.984339

[189] Rikmann E, Zekker I, Tomingas M, Vabamae P, Kroon K, Saluste A, et al. Comparison of sulfate-reducing and conventional Anammox upflow anaerobic sludge blanket reactors. J Biosci Bioeng. 2014;118(4):426–33. Epub 2014/05/28. doi: 10.1016/j.jbiosc.2014.03.012 . 24863179

[190] Liu S, Yang F, Gong Z, Meng F, Chen H, Xue Y, et al. Application of anaerobic ammonium-oxidizing consortium to achieve completely autotrophic ammonium and sulfate removal. Bioresour Technol. 2008;99(15):6817–25. Epub 2008/03/18. doi: 10.1016/j.biortech.2008.01.054 . 18343660

[191] Cai J, Jiang J, Zheng P. Isolation and identification of bacteria responsible for simultaneous anaerobic ammonium and sulfate removal. Sci China Chem. 2010;53(3):645–50. doi: 10.1007/s11426-010-0053-8

[192] Li G-X, Li H, Xiao K-Q, Bao P. Thiosulfate reduction coupled with anaerobic ammonium oxidation by Ralstonia sp. GX3-BWBA. ACS Earth Space Chem. 2020;4(12):2426–34. doi: 10.1021/acsearthspacechem.0c00267

