# Supplementary figures and images for: Giant sulfur bacteria (Beggiatoaceae) from sediments underlying the Benguela upwelling system host diverse microbiomes
Source: PLoS One. 2021 Nov 24;16(11):e0258124. doi: 10.1371/journal.pone.0258124 (PMC8612568; doi:10.1371/journal.pone.0258124)

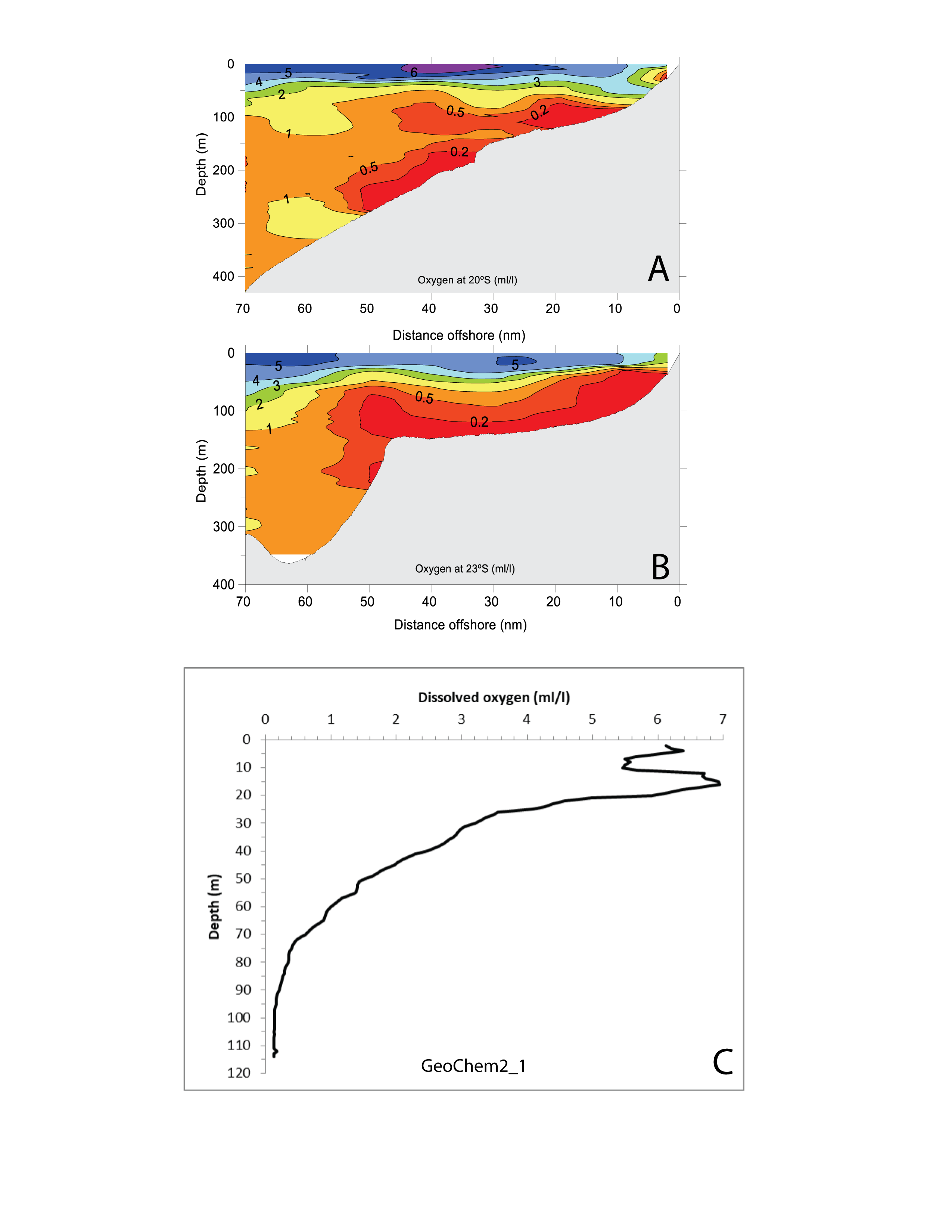

Supplement: S1 Fig — A. Along the -20 latitude, B. Along the -23 latitude, C. At Marine Station Geochem2_1 April 2017. (TIF) [file pone.0258124.s001.tif]

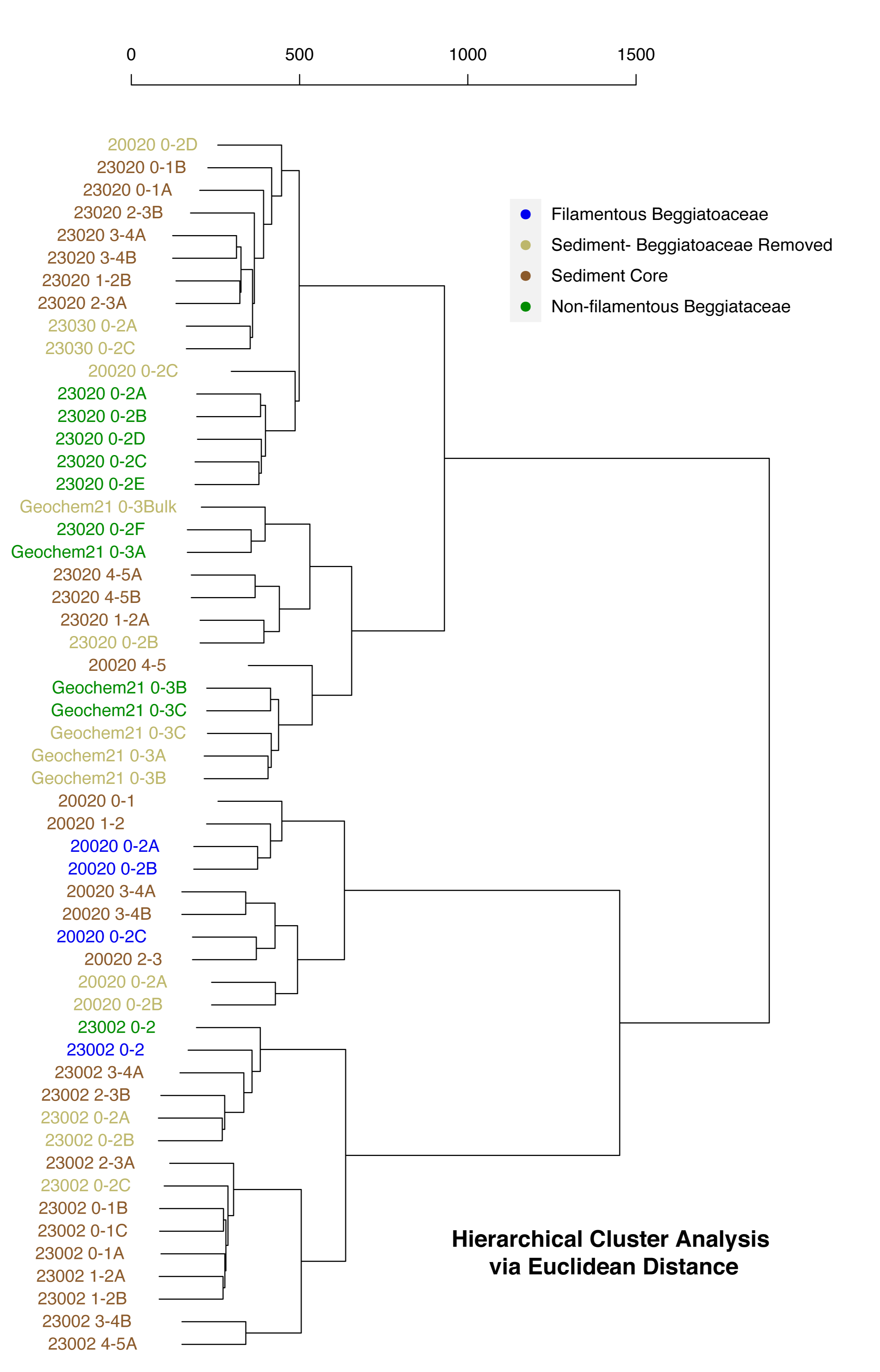

Supplement: S2 Fig — (TIF) [file pone.0258124.s002.tif]

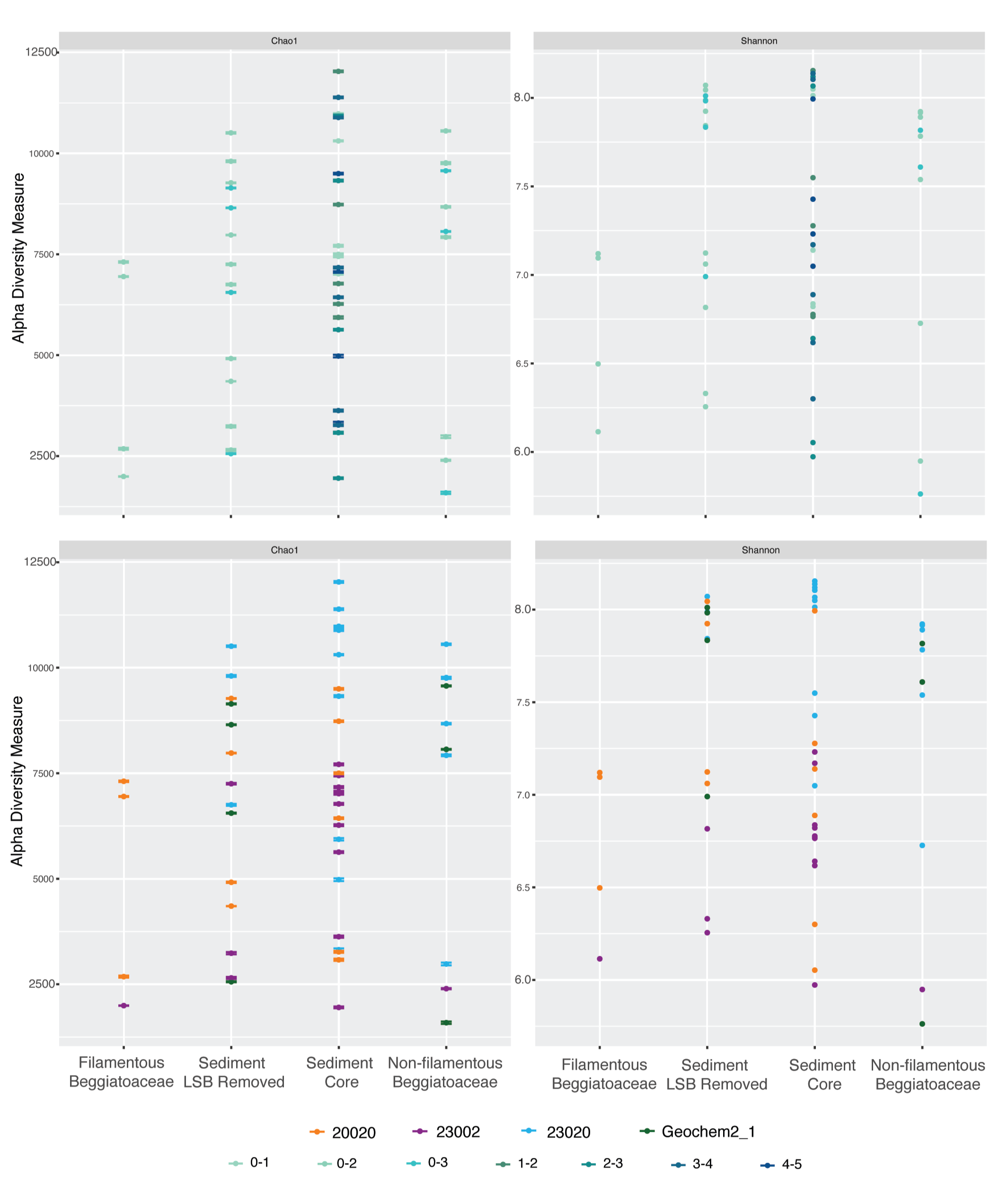

Supplement: S3 Fig — (TIF) [file pone.0258124.s003.tif]

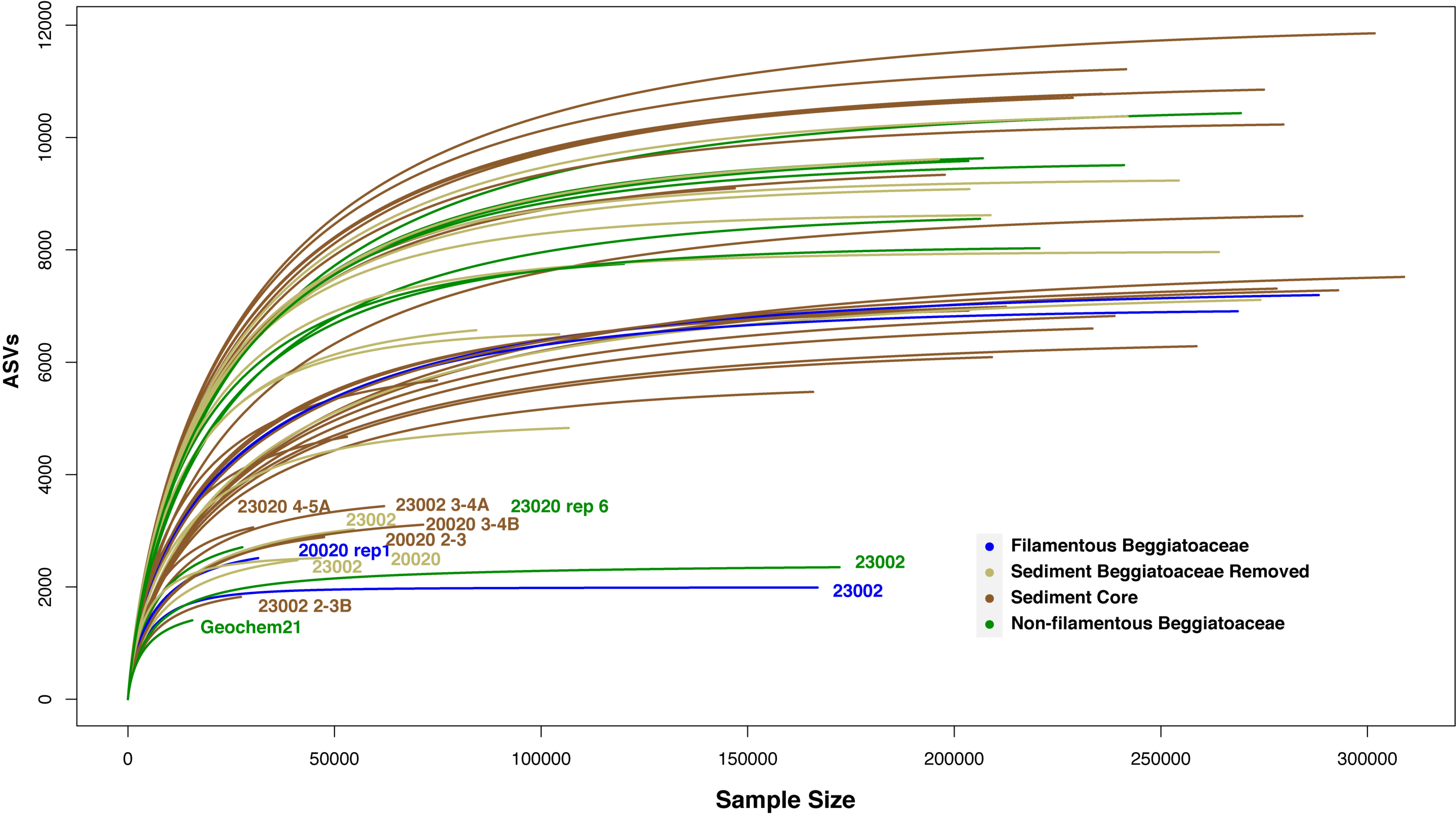

Supplement: S4 Fig — The plots demonstrate that some samples had a small library size which failed to adequate capture the entire community, which likely resulted in under detection via DESeq2 of some significant associations between host Beggiatoaceae and their attached epibionts. (TIF) [file pone.0258124.s004.tif]

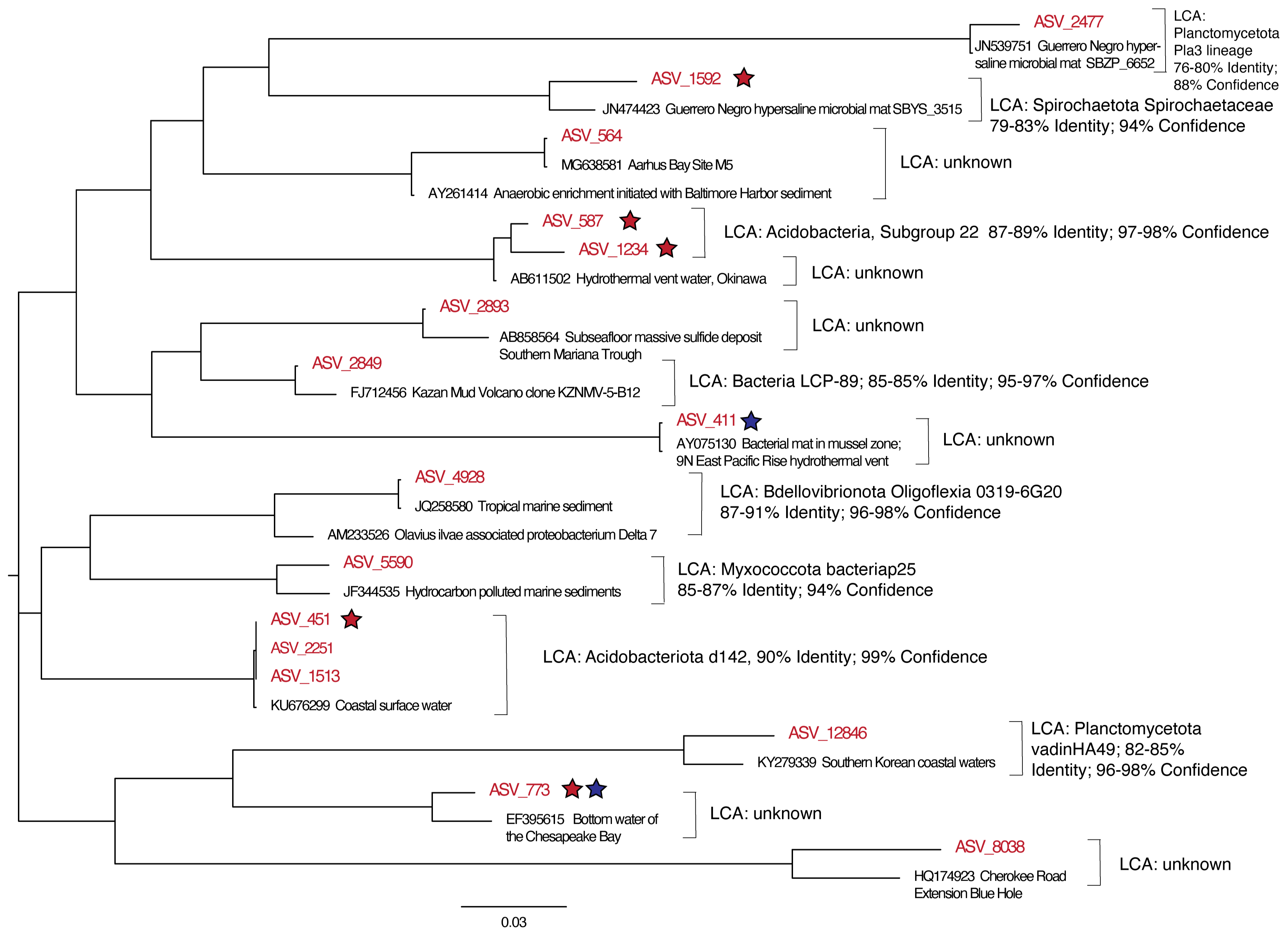

Supplement: S5 Fig — The 16S rRNA gene sequences were classified against the Silva database using the SINA aligner 1.2.1.1 with a 70% identity cutoff. Red stars indicated that the ASV was statistically significant for both stations 23020 and Geochem2_1 samples. Blue stars indicate that the ASV was likely associated with the non-filamentous LSB sample from Marine Station 23002. (TIF) [file pone.0258124.s005.tif]
