# Supplementary material for: Giant sulfur bacteria (Beggiatoaceae) from sediments underlying the Benguela upwelling system host diverse microbiomes
Source: PLoS One. 2021 Nov 24;16(11):e0258124. doi: 10.1371/journal.pone.0258124 (PMC8612568; doi:10.1371/journal.pone.0258124)
Supplement: S1 Text — (DOCX) [file pone.0258124.s006.docx]

Geochemical profiles of the top 10 cm of the sediment core collected from Station 23002 indicated that these sediments were experiencing extensive sulfate reduction at the surface of these sediments, Figure 2. Shifts in dominant taxa that may be associated with major metabolic regimes were evident at discreet horizons. Box plots of the most abundant phyla and proteobacterial classes, Figure 4, indicate that the community composition of the top 5 cm of the sediment cores contained a larger fraction of Caldatribacteriota, Cyanobacteria (predominantly diatom chloroplasts but free-living Cyanobacteria cannot be ruled out [1]) and Campilobacterota (formerly known as Epsilonproteobacteria) than other cores. The Verrucomicrobiota and ASVs unclassified at the phylum level composed a smaller fraction of the core community than the other marine stations. Caldatribacteriota is a newly named phylum that includes the former candidate phylum Atribacteria (OP9/JS1), which are thought to be primarily fermenters [2] that may have a syntrophic metabolism with methanogens [3] and that are commonly found in abundance in organic-rich anoxic marine sediments. Fermentation, such as that carried out by representatives of the Caldatribacteriota, is potentially the primary metabolism responsible for the increase in ammonium at depth [4] along with dissimilatory reduction of nitrate [5, 6]. Between 4-5 cm sediment depth, 1 mM of ammonium was consumed concomitantly with thiosulfate and sulfide, generating an increase in sulfate. Around 7 cm of depth, there was a significant drawdown of sulfate. A previous study of nearby sediments indicate that drawdown of sulfate is coupled to AOM [7]. We did not perform iTag sequencing on sediments below 5 cm. But ASVs of ANME-1a, ANME-1b and ANME-2c archaeal anaerobic methylotrophs were present throughout the top 5 cm of the core along with abundant and diverse archaeal methanogens.

One reasonable interpretation of these geochemical profiles is that the horizon at 4-5 cm reflects intense anammox activity coupled to sulfur oxidation and nitrate reduction [8, 9]. However, our iTag data indicated that the anammox bacteria in these sediments, *Ca*. Scalindua spp., were abundant primarily in the top 3 cm and were almost absent between 4-5 cm of depth. Since anammox bacterial abundance has been shown to be directly correlated to anammox activity [10], it seems likely that other processes were removing NH_4_^+^ at this horizon. But our iTag data provided few clues as to the metabolic processes responsible for these geochemical profiles. For a more in-depth discussion, please see SI text.

Aerobic archaeal ammonia oxidizers, *Ca*. Nitrosopelagicus and *Ca*. Nitrosopumilus, were present in these sediments, but not at the 4-5 cm horizon. Also present were bacterial denitrifying methylotrophs, ASVs of Methylomonadaceae, Methyloligellaceae, and *Sedimentitalea* [11, 12]. Some aerobic methylotrophs are capable of ammonium oxidation [13] but there was no indication of enrichment of these strains at the 4-5 cm horizon. In the top 3 cm most of the Beggiatoaceae ASVs and many other sulfide-oxidizers were present, esp. those associated with DNRA. If ammonium oxidation was coupled to sulfur-oxidation in the top 3 cm, the activity was insufficient in consuming all of the reactants except perhaps nitrite, which was below the detection limit in these sediments.

The iTag data provided few other clues as to the origin of the oxidation/removal of ammonium between 4-5 cm of depth. One might expect that such a significant drawdown of substrates would result in the proliferation of the responsible actors at that horizon. But, we identified no single ASV or clade of ASVs meeting such criteria. There was a community shift at the 4-5 cm horizon, to greater diversity, Figure S2, and a lower abundance of ASVs. Likely sulfur-oxidizers moderately enriched at the 3-5 cm horizons include an unclassified Thiotrichaceae ASV and the cable bacterium *Ca*. Electrothrix. There were more ASVs of unclassified Planctomycetes at the 4-5cm horizon than in the horizons above. The most significant shift in individual ASVs, as well as clades of ASVs, were associated with certain diatom chloroplasts ASVs for which a blastN query against the NCBI non-redundant nucleotide collection revealed they were likely endosymbiotic plastids in benthic foraminifera [14]. A few of these ASVs were some of the most abundant ASVs above the 4-5 cm horizon and dropped in concentration by 2 orders of magnitude at the 4-5 cm horizon. But one ASV was enriched at the 4-5 cm horizon, which was a 100% match to a plastid of *Virgulinella fragilis* (JN207229, Walvis Bay, Namibia)[14], a foraminifera once thought to be symbiotic with members of the Beggiatoaceae [15]. Benthic foraminifera migrate in anoxic sediments and are known to host sulfur-cycling bacterial endobionts [16], store and denitrify nitrate [17, 18] and possibly store ammonium as well [19, 20]. Thus, it is conceivable that foraminifera play a significant role in the drawdown of ammonium at this depth. Alternatively, the top 3 cm of the core were sampled with a syringe because it was “soupy” in texture while the lower sections of the core were subsampled with a knife. Perhaps this significant change is sediment cohesion coupled with the reduction of competition with most of the benthic forams for substrates and/or the reduction of DNRA in the 4-5 cm horizon were significant enough changes to result in greater depletion of ammonium by anammox bacteria in low abundances at this horizon. But alternative and largely understudied processes that consume high levels of ammonium anaerobically do exist. Anaerobic lithotrophic ammonium oxidation could also be coupled with either iron (ferromox), sulfate (sulfammox) or thiosulfate (sammox) respiration [21] and/or heterotrophic nitrification as well as nitrifier denitrification could be occurring as well [22].

**References**

1. Puente-Sanchez F, Arce-Rodriguez A, Oggerin M, Garcia-Villadangos M, Moreno-Paz M, Blanco Y, et al. Viable cyanobacteria in the deep continental subsurface. Proc Natl Acad Sci U S A. 2018;115(42):10702-7. Epub 2018/10/03. doi: 10.1073/pnas.1808176115. PubMed PMID: 30275328; PubMed Central PMCID: PMCPMC6196553.

2. Nobu MK, Dodsworth JA, Murugapiran SK, Rinke C, Gies EA, Webster G, et al. Phylogeny and physiology of candidate phylum ‘Atribacteria’ (OP9/JS1) inferred from cultivation-independent genomics. ISME J. 2016;10(2):273-86. Epub 2015/06/20. doi: 10.1038/ismej.2015.97. PubMed PMID: 26090992; PubMed Central PMCID: PMCPMC4737943.

3. Lee YM, Hwang K, Lee JI, Kim M, Hwang CY, Noh H-J, et al. Genomic insight Into the predominance of candidate phylum Atribacteria JS1 lineage in marine sediments. Front Microbiol. 2018;9:2909. Epub 2018/12/18. doi: 10.3389/fmicb.2018.02909. PubMed PMID: 30555444; PubMed Central PMCID: PMCPMC6281690.

4. Jacobson ME, Mackin JE, Capone DG. Ammonium production in sediments inhibited with molybdate: Implications for the sources of ammonium in anoxic marine sediments. Appl Environ Microb. 1987;53(10):2435-9. Epub 1987/10/01. doi: 10.1128/AEM.53.10.2435-2439.1987. PubMed PMID: 16347462; PubMed Central PMCID: PMCPMC204125.

5. Kraft B, Tegetmeyer HE, Sharma R, Klotz MG, Ferdelman TG, Hettich RL, et al. The environmental controls that govern the end product of bacterial nitrate respiration. Science. 2014;345(6197):676-9. Epub 2014/08/12. doi: 10.1126/science.1254070. PubMed PMID: 25104387.

6. Devol AH. Denitrification, anammox, and N(2) production in marine sediments. Ann Rev Mar Sci. 2015;7:403-23. Epub 2015/01/07. doi: 10.1146/annurev-marine-010213-135040. PubMed PMID: 25560607.

7. Brüchert V, Currie B, Peard KR, Lass U, Endler R, Dübecke A, et al. Biogeochemical and physical control on shelf anoxia and water column hydrogen sulphide in the Benguela coastal upwelling system off Namibia. Past and present water column anoxia: Springer; 2006. p. 161-93.

8. Prokopenko MG, Hirst MB, De Brabandere L, Lawrence D, Berelson W, Granger J, et al. Nitrogen losses in anoxic marine sediments driven by *Thioploca*-anammox bacterial consortia. Nature. 2013;500(7461):194-8. Epub 2013/08/09. doi: 10.1038/nature12365. PubMed PMID: 23925243.

9. Rios-Del Toro EE, Cervantes FJ. Coupling between anammox and autotrophic denitrification for simultaneous removal of ammonium and sulfide by enriched marine sediments. Biodegradation. 2016;27(2-3):107-18. Epub 2016/03/21. doi: 10.1007/s10532-016-9759-4. PubMed PMID: 26994921.

10. Pajares S, Ramos R. Processes and microorganisms involved in the marine nitrogen cycle: Knowledge and gaps. Front Mar Sci. 2019;6. doi: 10.3389/fmars.2019.00739.

11. Vekeman B, Kerckhof FM, Cremers G, de Vos P, Vandamme P, Boon N, et al. New M*ethyloceanibacter* diversity from North Sea sediments includes methanotroph containing solely the soluble methane monooxygenase. Environ Microbiol. 2016;18(12):4523-36. Epub 2016/08/09. doi: 10.1111/1462-2920.13485. PubMed PMID: 27501305.

12. Orata FD, Kits KD, Stein LY. Complete genome sequence of *Methylomonas denitrificans* strain FJG1, an obligate aerobic methanotroph that can couple methane oxidation with denitrification. Genome Announc. 2018;6(17).

13. Versantvoort W, Pol A, Jetten MSM, van Niftrik L, Reimann J, Kartal B, et al. Multiheme hydroxylamine oxidoreductases produce NO during ammonia oxidation in methanotrophs. Proc Natl Acad Sci U S A. 2020;117(39):24459-63. Epub 2020/09/12. doi: 10.1073/pnas.2011299117. PubMed PMID: 32913059; PubMed Central PMCID: PMCPMC7533708.

14. Tsuchiya M, Toyofuku T, Uematsu K, Brüchert V, Collen J, Yamamoto H, et al. Cytologic and genetic characteristics of endobiotic bacteria and kleptoplasts of *Virgulinella fragilis* (Foraminifera). J Eukaryot Microbiol. 2015;62(4):454-69. Epub 2014/12/17. doi: 10.1111/jeu.12200. PubMed PMID: 25510528.

15. Erbacher J, Nelskamp S. Comparison of benthic foraminifera inside and outside a sulphur-oxidizing bacterial mat from the present oxygen-minimum zone off Pakistan (NE Arabian Sea). Deep Sea Research Part I: Oceanographic Research Papers. 2006;53(5):751-75.

16. Salonen IS, Chronopoulou P, Bird C, Reichart G, Koho KA. Enrichment of intracellular sulphur cycle -associated bacteria in intertidal benthic foraminifera revealed by 16S and aprA gene analysis. Sci Rep. 2019;9(1):11692. Epub 2019/08/14. doi: 10.1038/s41598-019-48166-5. PubMed PMID: 31406214; PubMed Central PMCID: PMCPMC6690927.

17. Glock N, Roy AS, Romero D, Wein T, Weissenbach J, Revsbech NP, et al. Metabolic preference of nitrate over oxygen as an electron acceptor in foraminifera from the Peruvian oxygen minimum zone. Proc Natl Acad Sci U S A. 2019;116(8):2860-5. Epub 2019/02/08. doi: 10.1073/pnas.1813887116. PubMed PMID: 30728294; PubMed Central PMCID: PMCPMC6386669.

18. Koho KA, Piña-Ochoa E, Geslin E, Risgaard-Petersen N. Vertical migration, nitrate uptake and denitrification: survival mechanisms of foraminifers (*Globobulimina turgida*) under low oxygen conditions. FEMS Microbiol Ecol. 2011;75:273–83.

19. Nomaki H, Bernhard JM, Ishida A, Tsuchiya M, Uematsu K, Tame A, et al. Intracellular isotope localization in *Ammonia* sp. (Foraminifera) of oxygen-depleted environments: results of nitrate and sulfate labeling experiments. Front Microbiol. 2016;7:163. Epub 2016/03/01. doi: 10.3389/fmicb.2016.00163. PubMed PMID: 26925038; PubMed Central PMCID: PMCPMC4759270.

20. LeKieffre C, Jauffrais T, Geslin E, Jesus B, Bernhard JM, Giovani ME, et al. Inorganic carbon and nitrogen assimilation in cellular compartments of a benthic kleptoplastic foraminifer. Sci Rep. 2018;8(1):10140. Epub 2018/07/06. doi: 10.1038/s41598-018-28455-1. PubMed PMID: 29973634; PubMed Central PMCID: PMCPMC6031614.

21. Rios-Del Toro EE, Cervantes FJ. Anaerobic ammonium oxidation in marine environments: contribution to biogeochemical cycles and biotechnological developments for wastewater treatment. Reviews in Environmental Science and Bio/Technology. 2019;18(1):11-27. doi: 10.1007/s11157-018-09489-3.

22. Stein LY. Heterotrophic nitrification and nitrifier denitrification. Nitrification. 2011:95-114.
