# Supplementary material for: Giant sulfur bacteria (Beggiatoaceae) from sediments underlying the Benguela upwelling system host diverse microbiomes
Source: PLoS One. 2021 Nov 24;16(11):e0258124. doi: 10.1371/journal.pone.0258124 (PMC8612568; doi:10.1371/journal.pone.0258124)
Supplement: Attachment — Submitted filename: Response2revewiers_PLOS.docx [file pone.0258124.s008.docx]

Response to Reviewers: PLOS ONE Decision: Revision required [PONE-D-21-15605] - [EMID:f3dd6ffaca44656e]

Dear Editor and Reviewers,

Thank you for taking the time to review our manuscript and your thoughtful comments. We added you to the Acknowledgements section:

**“Lastly, we thank the reviewers and editor for their thoughtful insight.”**

Also, we made one additional change in the manuscript. In the conclusions, 132 was changed to the correct number 123. Please note, that using Marked-up in Microsoft Word has altered the line numbers (some line numbers are missing, like lines 41 & 42). Below are our responses to your specific comments.

***Response to Editor***

*I suggest a minor editorial comment: on line 31, I think it would be better to say that they were "pooled by morphotype for community analysis" or something similar; the goal of the iTag sequencing is more important than the approach.*

**Corrected to:** To elucidate community members that were directly attached and enriched in both filamentous Beggiatoaceae, namely *Ca*. Marithioploca spp. and *Ca*. Maribeggiatoa spp., as well as non-filamentous Beggiatoaceae, *Ca.* Thiomargarita spp**., the Beggiatoaceae were pooled by morphotype for community analysis**.

***Response to Reviewer #1***

*L. 88: i.e. should not be written in italic. Commas after Beggiatoa spp. and Ca. Isobeggiatoa spp. are needed.* **Corrected**.

*Please, delete comments from S1 Text.* **Corrected.**

*L. 266: ‘Thioflexithrix’, not ‘Thioflexothrix’. It is interesting that ASVs from marine sediment can be classified as Thioflexithrix, because originally described Thioflexithrix psekupsensis was isolated from freshwater habitat. it might be worth discussing this fact.*

**Added: To the best of our knowledge, this is the first report of a *Thioflexithrix* from a marine environment. The recently discovered type strain, *T. psekupsensis* D3, was isolated from a thermal sulfidic spring (37).**

*L. 485: it may be worth mentioning that the opportunity of Beggiatoaceae association with Spirochaeta was already shown (Grabovich et al., 2021 DOI: 10.1016/j.biosystems.2020.104322)*

**Thanks for catching this! We researched this further and found that there have been observations of such associations going back to 1910! Unfortunately, the 1910 & 1912 manuscripts are not available; but, they were referenced in the Dyar 1947 manuscript (referenced below: Dyar, 1947; Blakemore & Canale-Parola, 1973; and Grabovich et al., 2021).**

**Added:** “**Most of these clades are poorly studied, but there have been many observations of morphologically-distinct members of the Spirochaetota in association with the Beggiatoaceae (120-122).** Many of these **clades** likely …”. (changed to “clades” in the next sentence because “strains” might infer just the Spirochetes vs. all of the clades discussed above).

*L. 521: Thiomargarita should be written in italic.* **Corrected.**

*Ref 25. Leptomitoformis should be written with a lowercase letter.* **Corrected.**

***Response to Reviewer #2.***

*line 91: "way" should be "away" (I believe).* **Corrected to “away”.** *line 98: remove "But".* **Corrected.** *line 122: remove "But"* **Corrected.** *line 132: add hyphen to make it "Beggiatoaceae-dominated" line 133: remove hyphen to make it "sulfate reduction"* **Corrected.**

*lines 272-274: Is "introns" the correct term here? I was not aware that introns were found in prokaryote genomes. Also, "which is" should be changed to "which are," if you are referring to multiple introns.* **Corrected to “which are”.**

**Yes, “intron” is the correct term. Although rare in prokaryotes, introns do occur, particularly in 16S rRNA genes but also occasionally in functional genes. Introns contributed to missing some candidate phyla by classic 16S rRNA gene sequencing. Genomes of marine strains of the Beggiatoaceae often contain both Group I and Group II introns (below references: Salman et al., 2012, Flood, et al., 2016).**

*lines 438-450: "Significantly associated" should be further defined here. How does DESeq2 make this determination (I assume with statistical analyses but if so, which ones).*

**Added the following. “DESeq2 is an R package that was original designed for detecting differential expression between two treatments in RNA sequencing experiments and accounts for differences in sample library sizes. Significant associations were determined through a Wald’s Test (p-value > 0.01) adjusted for potential false discoveries (padj).”**

*lines 525-527: This sentence is somewhat confusing- consider rephrasing.*

**Corrected to: “Some ASVs associated with non-filamentous Beggiatoaceae were in relatively low abundances, i.e.** at Station 23020 an ASV of Desulfocaspa and an unclassified Desulfobulbales and at Station GeoChem2_1, an ASV of Desulfuromusa.

*line 534: remove "But"* **Corrected.** *line 535: change "thus" to "and thus"* **Corrected.** *line 555: "one ASV" but then two orders are named. Does this mean one ASV from each order?*

**Corrected to**: In addition, **two** ASV**s** of the Alphaproteobacteria clades Rhizobiales and Rhodobacterales could represent organoheterotrophic sulfur-oxidizers.

*line 706: replace comma after "bacteria" with a colon.* **Corrected.**

***References***

Blakemore, RP, and E Canale-Parola. "Morphological and Ecological Characteristics of *Spirochaeta Plicatilis*." *Archiv für Mikrobiologie* 89, no. 4 (1973): 273-89. <https://dx.doi.org/https://doi-org.ezp2.lib.umn.edu/10.1007/BF00408895>.

Dyar, MT. "Isolation and Cytological Study of a Free-Living Spirochete." *Journal of Bacteriology* 54, no. 4 (1947): 483-93. <https://dx.doi.org/https://doi.org/10.1128/jb.54.4.483-493.1947>.

Flood, Beverly E., Palmer Fliss, Daniel Seth Jones, Gregory J. Dick, Sunit Jain, Anne-Kristin Kaster, Matthias Winkel, Marc Mußmann, and Jake Bailey. "Single-Cell (Meta-)Genomics of a Dimorphic *Candidatus* Thiomargarita Nelsonii Reveals Genomic Plasticity." Original Research, *Frontiers in Microbiology* 7 (2016-May-3 2016): 603. <https://dx.doi.org/10.3389/fmicb.2016.00603>.

Grabovich, M. Y., M. V. Gureeva, and G. A. Dubinina. "The Role of the "*Thiodendron*" Consortium in Postulating the Karyomastigont Chimaera of the Endosymbiosis Theory by Lynn Margulis." *Biosystems* 200 (Feb 2021): 104322. <https://dx.doi.org/10.1016/j.biosystems.2020.104322>.

Salman, Verena, Rudolf Amann, David A. Shub, and Heide N. Schulz-Vogt. "Multiple Self-Splicing Introns in the 16s Rrna Genes of Giant Sulfur Bacteria." *Proceedings of the National Academy of Sciences* 109, no. 11 (Mar 13 2012): 4203–08. <https://dx.doi.org/10.1073/pnas.1120192109>.
